# Mapping the density of giant trees in the Amazon

**DOI:** 10.1111/nph.70634

**Published:** 2025-10-14

**Authors:** Robson Borges de Lima, Diego Armando Silva da Silva, Matheus Henrique Nunes, Paulo R. de Lima Bittencourt, Peter Groenendyk, Cinthia Pereira de Oliveira, Daniela Granato‐Souza, Rinaldo L. Caraciolo Ferreira, José A. Aleixo da Silva, Jesús Aguirre‐Gutiérrez, Toby Jackson, João R. de Matos Filho, Perseu da Silva Aparício, Joselane P. Gomes da Silva, José Julio de Toledo, Marcelino Carneiro Guedes, Danilo R. Alves de Almeida, Niro Higuchi, Fabien H. Wagner, Jean Pierre Ometto, Eric Bastos Görgens

**Affiliations:** ^1^ Laboratório de Manejo Florestal Universidade do Estado do Amapá 68901‐262 Macapá AP Brazil; ^2^ Instituto Federal de Educação Ciência e Tecnologia do Amapá 68.920‐000 Laranjal do Jari AP Brazil; ^3^ Department of Geographical Sciences University of Maryland College Park MD 20742 USA; ^4^ School of Earth and Environment Sciences Cardiff University CF10 3AT Cardiff UK; ^5^ Department of Plant Biology Institute of Biology, University of Campinas PO Box 6109 13083‐970 Campinas SP Brazil; ^6^ Department of Natural Resources and Environmental Sciences College of Agricultural Alabama A & M University Huntsville 35762 AL USA; ^7^ Departamento de Ciência Florestal Universidade Federal Rural de Pernambuco 52171‐900 Recife PE Brazil; ^8^ Environmental Change Institute University of Oxford Oxford OX13QY UK; ^9^ School of Biological Sciences University of Bristol Bristol BS8 1QU UK; ^10^ Promotoria de Justiça do Meio Ambiente Conflitos Agrários, Ministério Público 68903‐883 Macapá AP Brazil; ^11^ Universidade Federal do Amapá 68903‐419 Macapá AP Brazil; ^12^ Empresa Brasileira de Pesquisa Agropecuária 68903‐419 Macapá AP Brazil; ^13^ Department of Forest Sciences ‘Luiz de Queiroz’ College of Agriculture, University of São Paulo 13418‐900 Piracicaba SP Brazil; ^14^ Instituto Nacional de Pesquisas da Amazônia, Coordenação de Pesquisas em Silvicultura Tropical 69080‐971 Manaus AM Brazil; ^15^ Jet Propulsion Laboratory, California Institute of Technology 4800 Oak Grove Pasadena CA 91109 USA; ^16^ Instituto Nacional de Pesquisas Espaciais 12227‐010 São José dos Campos SP Brazil; ^17^ Departamento de Engenharia Florestal Universidade Federal dos Vales do Jequitinhonha e Mucuri 39100‐000 Diamantina MG Brazil

**Keywords:** airborne LiDAR, biogeographic provinces, environmental factors, spatial modeling, tallest trees

## Abstract

- Tall trees (height ≥ 60 m) are keystone elements of tropical forests, strongly influencing biodiversity, carbon storage, and ecosystem resilience. Yet, their density and spatial distribution remain poorly quantified, especially in remote Amazonian regions, limiting our understanding of their ecological roles and contribution to forest–climate interactions.
- We combined airborne LiDAR data from 900 transects across the Brazilian Amazon with environmental predictors to model tall‐tree density. Spatial extrapolations allowed us to generate regional distribution estimates and assess associations with climate, topography, and disturbance regimes.
- Our model predicts that tall trees are unevenly distributed, with *c.* 14% of the estimated density concentrated in *c.* 1% of the Amazon and *c.* 50% within *c.* 11%. The highest densities occur in Roraima and the Guiana Shield provinces, where water availability is high and lightning or storm incidence is low. Modeled density strongly correlates with aboveground biomass, highlighting the disproportionate contribution of tall trees to carbon stocks. We estimate *c.* 55.5 million tall trees across the Brazilian Amazon.
- These findings demonstrate that tall‐tree distribution is a crucial but underused predictor for biomass models. Understanding their ecological and spatial dynamics is vital for forest conservation and climate‐resilience strategies under increasing anthropogenic pressures.

## Resumo

- Árvores altas (altura ⩾ 60 m) são elementos‐chave das florestas tropicais, influenciando fortemente a biodiversidade, o armazenamento de carbono e a resiliência dos ecossistemas. No entanto, sua densidade e distribuição espacial permanecem mal quantificadas, especialmente em regiões remotas da Amazônia, limitando nossa compreensão de seus papéis ecológicos e contribuição para as interações floresta‐clima.
- Combinamos dados LiDAR aéreos de 900 transectos na Amazônia brasileira com preditores ambientais para modelar a densidade de árvores altas. Extrapolações espaciais nos permitiram gerar estimativas de distribuição regional e avaliar associações com clima, topografia e regimes de perturbação.
- Nosso modelo prevê que as árvores altas estão distribuídas de forma desigual, com ~14% da densidade estimada concentrada em ~1% da Amazônia e ~50% em ~11%. As maiores densidades ocorrem em Roraima e no Escudo das Guianas, onde a disponibilidade hídrica é alta e a incidência de raios ou tempestades é baixa. A densidade modelada correlaciona‐se fortemente com a biomassa acima do solo (BPA), destacando a contribuição desproporcional das árvores altas para os estoques de carbono. Estimamos aproximadamente 55,5 milhões de árvores altas em toda a Amazônia brasileira.
- Estes resultados demonstram que a distribuição de árvores altas é um preditor crucial, mas subutilizado, para modelos de biomassa. Compreender sua dinâmica ecológica e espacial é vital para a conservação florestal e estratégias de resiliência climática sob crescentes pressões antrópicas.

## Resumen

- Los árboles altos (altura ⩾ 60 m) son elementos clave de los bosques tropicales, con una fuerte influencia en la biodiversidad, el almacenamiento de carbono y la resiliencia de los ecosistemas. Sin embargo, su densidad y distribución espacial siguen estando poco cuantificadas, especialmente en las regiones remotas de la Amazonía, lo que limita nuestra comprensión de sus funciones ecológicas y su contribución a las interacciones entre los bosques y el clima.
- Combinamos datos aerotransportados LiDAR de 900 transectos en la Amazonía brasileña con predictores ambientales para modelar la densidad de árboles altos. Las extrapolaciones espaciales nos permitieron generar estimaciones de distribución regional y evaluar las asociaciones con el clima, la topografía y los regímenes de perturbación.
- Nuestro modelo predice que los árboles altos tienen una distribución desigual, con aproximadamente el 14 % de la densidad estimada concentrada en aproximadamente el 1 % de la Amazonía y aproximadamente el 50 % en aproximadamente el 11 %. Las mayores densidades se encuentran en Roraima y el Escudo Guayanés, donde la disponibilidad de agua es alta y la incidencia de rayos o tormentas es baja. La densidad modelada presenta una fuerte correlación con la biomasa aérea (AGB), lo que destaca la contribución desproporcionada de los árboles altos a las reservas de carbono. Estimamos aproximadamente 55,5 millones de árboles altos en la Amazonia brasileña.
- Estos hallazgos demuestran que la distribución de árboles altos es un predictor crucial, aunque poco utilizado, para los modelos de biomasa. Comprender su dinámica ecológica y espacial es vital para la conservación forestal y las estrategias de resiliencia climática bajo la creciente presión antropogénica.

## Résumé

- Les grands arbres (hauteur ⩾ 60 m) sont des éléments clés des forêts tropicales, influençant fortement la biodiversité, le stockage du carbone et la résilience des écosystèmes. Pourtant, leur densité et leur répartition spatiale restent mal quantifiées, notamment dans les régions amazoniennes reculées, ce qui limite notre compréhension de leurs rôles écologiques et de leur contribution aux interactions forêt‐climat.
- Nous avons combiné des données LiDAR aéroportées provenant de 900 transects en Amazonie brésilienne avec des prédicteurs environnementaux afin de modéliser la densité des grands arbres. Les extrapolations spatiales nous ont permis d'estimer la répartition régionale et d'évaluer les associations avec le climat, la topographie et les régimes de perturbation.
- Notre modèle prédit une répartition inégale des grands arbres, avec environ 14 % de la densité estimée concentrée sur environ 1 % de l'Amazonie et environ 50 % sur environ 11 %. Les densités les plus élevées se trouvent dans le Roraima et le bouclier guyanais, où la disponibilité en eau est élevée et l'incidence de la foudre ou des orages est faible. La densité modélisée est fortement corrélée à la biomasse aérienne (BAH), soulignant la contribution disproportionnée des grands arbres aux stocks de carbone. Nous estimons qu'il y a environ 55,5 millions de grands arbres en Amazonie brésilienne.
- Ces résultats démontrent que la distribution des grands arbres est un prédicteur crucial, mais sous‐utilisé, pour les modèles de biomasse. Comprendre leur dynamique écologique et spatiale est essentiel pour la conservation des forêts et les stratégies de résilience climatique face aux pressions anthropiques croissantes.

## Introduction

Understanding the density and distribution of larger trees in the Amazon is vital for predicting the carbon balance of Amazonian ecosystems with global environmental change (Bastin *et al*., 2018; Lutz *et al*., 2018; Enquist *et al*., 2020). Particularly, the local and regional variations in the density of large trees are strongly linked to spatial variations in the aboveground biomass (AGB) of tropical forests (Slik *et al*., 2013; Birdsey *et al*., 2023) and regulate the microclimate, water availability, light intensity, and understory species diversity (Lindenmayer, 2017; Lindenmayer & Laurance, 2017; Brando, 2018; Pinho *et al*., 2020; Draper *et al*., 2021). Around 390 billion trees are estimated to inhabit the Amazon (Crowther *et al*., 2015). However, large uncertainties remain regarding how many tall trees reach the uppermost stratum of Amazonian forest canopies and how their survival is locally and regionally distributed. These large trees take centuries to reach such sizes, and many of the species may be unable to regenerate on timescales compatible with the planet's rapidly changing climate (Larjavaara, 2014; Bennett *et al*., 2015; Ali & Wang, 2021). Although there is still limited empirical evidence quantifying the recovery dynamics of giant trees across tropical forests, their exceptional longevity – combined with increasing forest degradation and intensifying climate‐related disturbances (e.g. drought, wind, lightning) – suggests that they are being lost at a faster rate than they are being replaced (Trenberth *et al*., 2014; McDowell *et al*., 2018; Gora *et al*., 2025). This imbalance makes them particularly vulnerable under current environmental pressures (Piovesan & Biondi, 2021). Monitoring the density of giant trees is therefore essential to understanding forest ecosystem stability, resilience, and long‐term carbon storage potential.

The density of tall trees is largely influenced by variables that determine their growth and survival rates, encompassing both genetic factors and environmental conditions, such as climate, soil, and topography, which shape their structural characteristics (Jucker *et al*., 2018; Caron *et al*., 2021; Mills *et al*., 2023; Ter Steege *et al*., 2023). Previous studies, such as Slik *et al*. (2013), have demonstrated that large‐diameter trees – often strongly correlated with extreme height – play a dominant role in explaining AGB variation across tropical forests. Moderate temperatures and increased light availability (i.e. providing more energy for growth), alongside low water stress, are factors that can favor a large density of tall trees (Stephenson, 1990; Madrigal‐González *et al*., 2023). However, the future survival of large trees may be threatened by climate change and land‐use changes. For example, large climatic and atmospheric oscillations may lead to physiological and mechanical instability in the structure of tall trees (Gora & Esquivel‐Muelbert, 2021; Jackson *et al*., 2021). The efficient vascular systems of tall trees are particularly vulnerable to prolonged droughts, which can lead to the collapse of water and nutrient transport (Barros *et al*., 2019; Araújo *et al*., 2024), and high turbulence caused by strong winds and a high incidence of lightning kills tall trees disproportionately (Gora *et al*., 2021; Feng *et al*., 2023). Therefore, the ability of Amazonian forests to support tall trees likely depends on a balance between environmental factors associated with resource availability that promote tree growth to its potential maximum size, alongside mechanisms that promote survival under vegetation disturbances (Gorgens *et al*., 2021). Analyzing the density of tall trees as a function of environmental determinants is crucial to understanding the Amazon's stability under increasing environmental pressures and climate change.

Field inventories have traditionally played a crucial role in mapping forest patterns and assessing tree diversity in the Amazon, offering essential insights into species composition and distribution (ter Steege *et al*., 2013, 2023; ForestPlots.net *et al*., 2021; De Lima *et al*., 2023). However, conventional approaches based on forest inventory plots provide limited spatial coverage for evaluating the density of tall trees. This limitation is largely due to the logistical and technical challenges of detecting these rare and spatially scattered individuals, which often require large sampling areas in remote and difficult‐to‐access regions (Harris *et al*., 2021; Carvalho *et al*., 2023). Although field inventories remain fundamental for understanding tree‐environment interactions and ecological mechanisms, they frequently fall short in capturing the broad‐scale spatial variability and true density of tall trees across the Amazon.

**Fig. 1:**
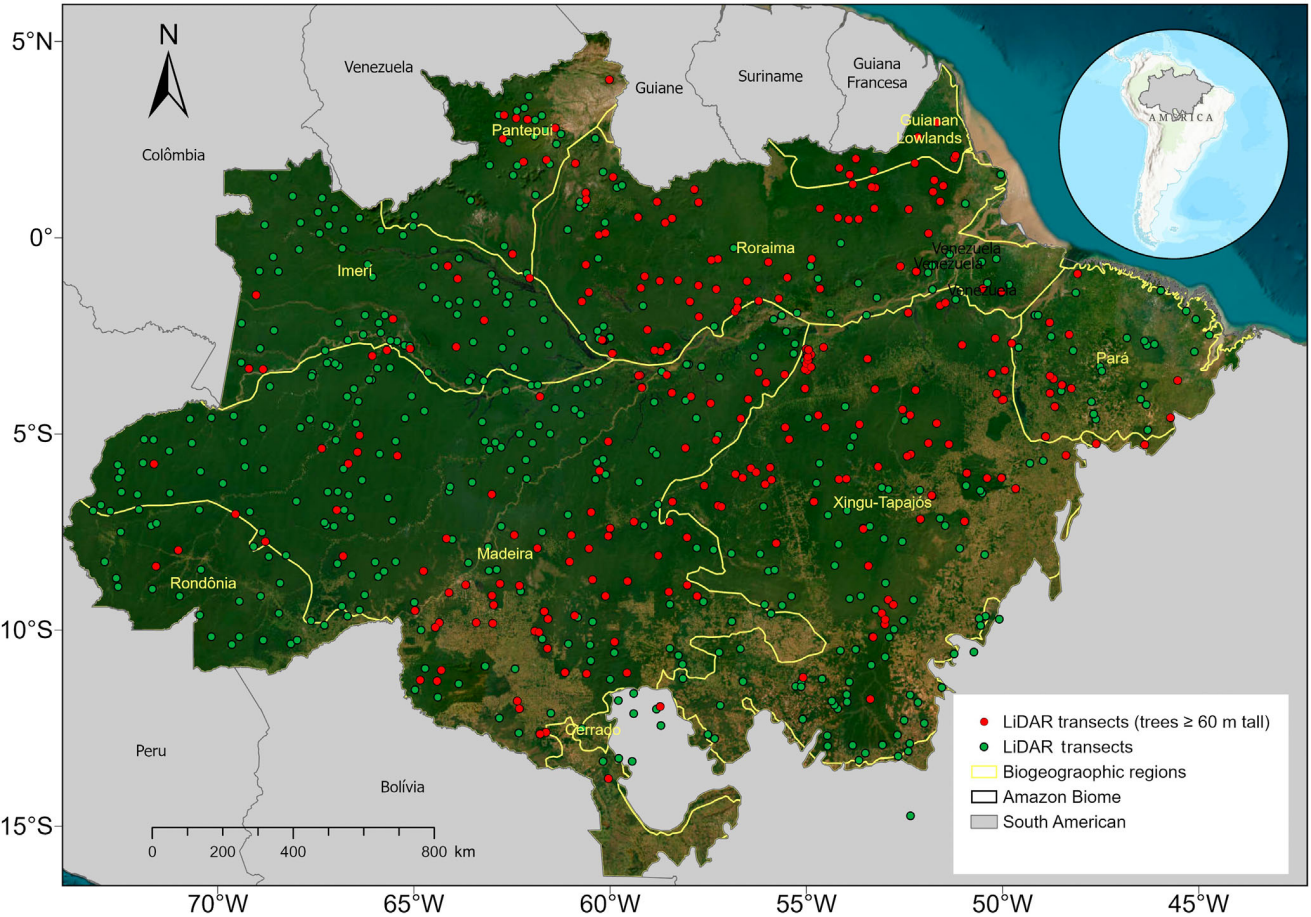
Distribution of transects mapped by airborne Light Detection and Ranging (LiDAR) across the Amazon Basin. The figure shows 900 randomly distributed transects covering the eight biogeographic provinces in the region. Red points represent transects where trees taller than 60 m were identified, whereas green points indicate transects without trees ≥ 60 m. Yellow lines delimit major biogeographic regions, including Imeri, Roraima, Guiana Shield, Pantepui, Madeira, Rondônia, Xingu‐Tapajós, and Pará. The LiDAR data transects are available at https://zenodo.org/records/4091222#.YDe7l2hKjIV.

The advent of Light Detection and Ranging (LiDAR) has transformed forest assessments by providing high‐resolution, three‐dimensional data on forest structure over large spatial scales. This technology can be used for rapid and accurate estimates of canopy height and AGB in remote regions (Asner *et al*., 2010; Dubayah *et al*., 2010; Coomes *et al*., 2017). The ‘Biomass Estimation in the Amazon’ (EBA) program, for example, mapped 900 transects, covering 375 ha each, which represents a remarkable increase of > 100‐fold in sampling capacity compared to traditional forest inventory plot methods (Fig. 1). This technological breakthrough has, for the first time in the Amazon, enabled more precise and accurate mapping of biomass stocks (Ometto *et al*., 2023) and reshaped our understanding of forest structure and the distribution of giant trees in the Amazon (Gorgens *et al*., 2019, 2021). In particular, Gorgens *et al*. (2019) identified the tallest tree ever recorded in the Amazon – a *Dinizia excelsa* reaching 88.5 m height in the eastern portion of the Roraima biogeographical province in Brazil. This discovery underscored the ecological importance of these towering giants and highlighted the critical role remote sensing techniques have in uncovering previously unknown aspects of tropical forest ecosystems. Despite these advances, there is still a dearth of LiDAR‐based estimates of large tree density across the tropics. Better estimates of large‐tree density are crucial to improving our understanding of the ecological processes and mechanisms driving tree–environment interactions, which are vital for predicting the carbon cycle dynamics and species vulnerability of Amazonian forests.

Here, we generated the first map of the spatial distribution of the giant tree density (height ≥ 60 m) in the Amazon basin, integrating spatially explicit climate, topography, atmosphere, and soil information. We fit a Random Forest (RF) machine learning model to predict the density of giant trees across the Brazilian Amazon, based on environmental and climatic variables. Then, we assessed the underlying environmental factors and identified the principal components that explain most of the variance in tall‐tree density in these biogeographic provinces. We also analyze the broader implications of these results for biomass estimates. These comprehensive analyses provide new insights into how different environmental variables influence tall‐tree density and provide crucial information for forest conservation and management policies in the Amazon.

## Materials and Methods

### LiDAR data collection and standardization

The point cloud from airborne LiDAR was acquired between 2016 and 2018 for 900 transects distributed throughout the Brazilian Amazon biome (Fig. 1, Supporting Information Fig. S1a,b). The data collection was part of the EBA project developed by National Institute for Space Research (Gorgens *et al*., 2021; Ometto *et al*., 2023). The sensor used was the LiDAR HARRIER 68i, coupled to a CESSNA model 206 aircraft. The scanning angle was 45°, and the flight altitude was *c*. 600 m. The point cloud is formed by 4 returns m^−2^. Each transect mapped 3.75 km^2^ (representing 12.5 × 0.3 km strips). See more details at https://zenodo.org/records/4091222#.YDe7l2hKjIV.

For each LiDAR transect, tall trees were identified using a local maxima detection algorithm applied to a canopy height model (CHM) derived from a normalized digital surface model and a digital terrain model (Silva *et al*., 2022). The CHM was generated at 1‐m resolution, and local maxima were identified using a 100‐m circular moving window, which helps to suppress noise and avoid the overdetection of treetops in dense canopy areas. Each treetop was assigned a geographic coordinate and its maximum height extracted from the CHM. To facilitate analysis, detected treetops were aggregated by transect and categorized into three height strata: < 40, ≥ 40 and < 60, and ≥ 60 m, with the latter defined as ‘giant trees’ for the purposes of this study (Fig. S1b). A unique string for each transect assigned as ID (transect number); the transect central coordinates (latitude (LAT) and longitude (LON)) and total area (in hectares) formed the initial dataframe for subsequent analyses (Dataset S1). To ensure consistency and maximum accuracy in the density data per transect and the final density maps, we standardized the area size to square kilometers (km^2^). Considering only tall trees (height ≥ 60 m), we detected their presence in 313 out of the 900 transects (*c*. 35%) across the Amazon biome. However, all 900 transects, including those with zero density of tall trees (*n* = 587), were used in the spatial modeling (RF) and principal component analysis (PCA). This approach ensured a comprehensive and representative analysis of the full gradient of environmental conditions and tall tree occurrence across the biome.

All transects in the final tree density data matrix cover eight biogeographic provinces proposed by Morrone (2014). This biogeographic definition seeks a universal classification in provinces with similar macroecological characteristics of biodiversity. To understand the optimal environmental conditions for the occurrence of the tallest trees, the density data km^−2^ were linked to spatially explicit environmental factors. The wide distribution of our sampling points (in number and distribution of LiDAR transects) provides a substantially representative sampling effort for this vegetation stratum. It ensures that any uncertainty in transect locations or minor changes in forest area in the Amazon region are unlikely to alter mean values or estimates of giant's tree density.

### Environmental factors

Our research initially involved 16 spatially explicit environmental predictor variables representing topography, climate, and soil, as detailed in Table 1 and Fig. S2. The data were then carefully cropped to fit the geographic boundaries of the Brazilian Amazon biome and, when necessary, resampled to a spatial resolution of 30 arc seconds (*c*. 1 km).

**Table 1:** List of the main environmental variables selected for this study.

Environmental variable					
Category	Sub‐category	Name and unit	Abbreviation	Spatial resolution (period)	Source
Topographic	Elevation	Elevation above sea level (m)	elev	30 m	SRTM
Climatic	Temperature	Mean annual temperature (°C)	tannual	30 arc seconds	WorldClim
		Maximum temperature (°C)	tmax	30 arc seconds	WorldClim
		Temperature seasonality (%)	tseason	30 arc seconds	WorldClim
	Precipitation	Average annual precipitation (mm)	pannual	30 arc seconds	WorldClim
		Precipitation seasonality (%)	pseason	30 arc seconds	WorldClim
		Precipitation of the wettest month	pwettest	30 arc seconds	WorldClim
	Physiologic influence	Number of clear days per year (days)	clearDays	500 m (2014–2018)	MODIS
		Days with precipitation > 20 mm (days)	days20	0.05° (2014–2018)	CHIRPS
		Potential evapotranspiration (mm yr^−1^)	pet	2.5 arc minutes (1990–2016)	TerraClimate
		Fraction of absorbed photosynthetically active radiation (%)	fapar	0.05° (2016–2018)	NOAA AVHRR
	Stressors	Lightning rate (flashes rate yr^−1^)	lightning	0.1°	LIS TRMM
		Meridional speed (N–S) (m s^−1^)	vspeed	0.25° (2014–2018)	ECM‐RWF
		Zonal speed (W–E) (m s^−1^)	uspeed	0.25° (2014–2018)	ECM‐RWF
Edaphic	Fraction of clay content	Soil structure physical properties water availability (%)	clayContent	250 m	SoilGrids
	Fraction of water content	Soil structure physical properties water availability (%)	waterContent	250 m	SoilGrids

Variable categories, subcategories, names and their corresponding units and abbreviations are shown. AVHRR, Advanced Very High‐Resolution Radiometer; CHIRPS, Climate Hazards Group InfraRed Precipitation with Station; LIS TRMM, Lightning Imaging Sensor instrument aboard the Tropical Rainfall Measurement Mission; MODIS, Moderate Resolution Imaging Spectroradiometer; NOAA, National Oceanic and Atmospheric Administration; SRTM, Shuttle Radar Topography Mission; WorldClim, global climate data.

Information on temperature and precipitation derived from 19 bioclimatic variables was obtained from WorldClim v.2 (Fick & Hijmans, 2017). Precipitation seasonality (BIO15) represents the coefficient of variation of monthly precipitation values and is used as an indicator of the intensity of the dry season. Temperature seasonality (BIO4) reflects the SD of monthly mean temperatures (×100). Higher values for both variables indicate greater climatic variability across the year. The average number of cloudless days throughout the year was obtained using surface reflectance products from the MODIS (Moderate Resolution Imaging Spectroradiometer) sensor. We used the Terra MOD09GA v.6 product, which estimates the MODIS surface spectral reflectance duly corrected for atmospheric conditions.

The annual average number of days with precipitation > 20 mm was calculated from the precipitation time series of the Climate Hazards Group InfraRed Precipitation with Station (CHIRPS) dataset (Funk *et al*., 2015). Potential evapotranspiration was derived from data provided by TerraClimate, which combines WorldClim climatological normals, Climatic Research Unit (CRU) Ts4.0, the 55‐yr Japanese Reanalysis (JRA‐55) data, and the Penman‐Monteith methodology. The fraction of absorbed photosynthetically active radiation (FAPAR) was obtained from the calibrated and corrected land surface reflectance product of the Advanced Very High‐Resolution Radiometer (AVHRR) of the National Oceanic and Atmospheric Administration (NOAA), providing information on the photosynthetic activity of plants (Baret *et al*., 2013).

Lightning frequency, associated with weather events and tree mortality (Gora *et al*., 2020), was obtained from the Lightning Imaging Sensor (LIS) instrument aboard the Tropical Rainfall Measurement Mission, provided by NASA's Earth Observing System's Global Hydrological Resources Center (EOSDIS). Lightning activity was represented by average annual flash rate (flashes yr^−1^), derived from LIS/TRMM data at *c*. 0.1° spatial resolution. The variable expresses the total number of lightning flashes per year per grid cell and is not normalized by area, as each value is associated with a fixed spatial extent defined by the sensor resolution. To represent long‐term wind disturbance patterns, we used 5‐yr (2014–2018) averages of the daily maximum wind speed components (*zonal‐uspeed* and *meridional‐vspeed*), calculated from hourly wind data derived from the ERA‐Interim reanalysis (ECM‐RWF). For each grid cell, the maximum wind speed for each day was identified from hourly records, and these daily maxima were then averaged across the 5‐yr period to produce a robust indicator of prevailing peak wind conditions. Previous studies indicate that winds are correlated with disturbances that result in tree mortality in the Amazon (Marra *et al*., 2014; Rifai *et al*., 2016).

Soil variables were obtained from the SoilGrids database (Hengl *et al*., 2017), which applies machine learning to a global compilation of soil profiles. From this dataset, we selected two variables relevant to forest structure: clay content (% of fine particles < 2 μm) and soil water content (% volume at field capacity at 30 cm depth). Both layers were used at a spatial resolution of 250 m.

All geospatial data were processed in ArcMap 10.1 software. We extracted all geospatial covariate values from raster datasets for transect location points using the *raster::extract* function from the R raster package (Hijmans *et al*., 2025) to construct a standardized plot‐level dataframe.

### Principal component analyses

To explore associations between environmental factors and understand the patterns governing giant tree density, we applied PCA to the normalized dataset of predictors. PCA was conducted separately for the entire Amazon biome and for each biogeographic province, allowing us to identify the main environmental gradients at both regional and local scales. The input variables were standardized to zero mean and unit variance before analysis to account for differences in measurement scales. Biplots of the first two principal components were generated using the factoextra R package (Kassambara & Mundt, 2020), providing a visual representation of how environmental variables covary and how these patterns may influence tall tree density in different parts of the biome.

### Spatial modeling

A RF model was used to model the density of tall trees from the environmental variables (Cutler & Wiener, 2022). This machine learning method detects global trends present in data using an ensemble strategy of decision trees to predict tree density in the uppermost strata of the Amazon rainforest canopy using the 16 environmental covariates. The RF algorithm applies the general bootstrap aggregation technique (bagging) with a modified tree learning algorithm that selects a random subset of the features at each candidate split in the learning process (Liang *et al*., 2022). Since a random subset of variables is chosen for each tree, the RF algorithm based on trained tree ensembles avoids overfitting (Calhoun *et al*., 2020). It circumvents potential multicollinearity issues (Genuer & Poggi, 2020) between the predictor variables.

For rigorous evaluation of the RF model, we employed the *k*‐fold cross‐validation method and spatial cross‐validation. First, the 900 sampled transects were randomly divided into *k* subsets (or *folds*) of approximately equal size. The randomized cross‐validation was of the k‐fold type, randomly dividing into *k* groups. In this procedure, *k* is defined as 15. For each *k* subset, the RF model is trained using *k*−1 subsets as the training set and the remaining subset as the test (or validation) set. This process was repeated 100 times with sample replacement to examine the accuracy of the estimated tree density values. After each round of training and validation, we calculated model performance metrics, such as root mean square error (RMSE) and coefficient of determination (*R* ^2^). RMSE is a measure of accuracy that reflects the average magnitude of prediction errors, while *R* ^2^ represents the proportion of variance explained by the model. Both provide a comprehensive assessment of model accuracy. These metrics are stored for each fold. At the end of the process, the metrics obtained in each *k* validation round are aggregated, usually by the average, to provide an overall estimate of the model performance. This average provides a more stable and reliable measure of model accuracy than a simple single split between training and testing.

A spatial cross‐validation methodology was employed to assess the predictive ability and spatial uncertainty of an RF model in estimating the density of tall trees (height ≥ 60 m) based on environmental variables. The spatial cross‐validation accounts for the spatial autocorrelation among sampling points, thereby minimizing the overestimation bias of model accuracy metrics due to the spatial proximity between training and test data. Initially, the data were organized in a spatial structure using the *sf* library (Pebesma *et al*., 2024), allowing for the manipulation and visualization of sample points with geographic coordinates. Ten folds were used for spatial cross‐validation, each representing a unique data partition. This division was conducted randomly and in a balanced manner, ensuring that each sample point was used exactly once as a test set. By contrast, the others were used for training, thus ensuring the generalization of the results and control over prediction variability. We acknowledge that there is ongoing debate regarding the balance between spatial independence and representativeness in training/testing splits, particularly in ecological studies involving complex environmental gradients. To address this, we examined residual spatial patterns separately to identify areas of potential model underperformance.

For each fold, the model was fitted using the *ranger* package (Wright *et al*., 2017), an efficient implementation of RF configured with 500 trees. The response variable was the density of tall trees (*#N_trees_h60_km2*). The explanatory variables included climatic, topographic, and edaphic environmental factors, carefully selected to capture spatial variability in environmental conditions. To avoid missing data issues (NA), the complete cases function was applied to the training data, ensuring that only complete records were used to fit the model. After fitting the model in each fold, predictions were made for the corresponding test set. These predictions were stored in a matrix, where each column represented the predictions of a specific fold. The uncertainty associated with the predictions was estimated by calculating the SD across folds for each sample point, creating an uncertainty map. This SD measure reflects the variability in predictions across different folds, indicating areas with greater or lesser confidence in the model estimates. The statistical metrics used to evaluate model performance included the RMSE and the coefficient of determination (*R* ^2^) of the tall tree density calculated for each fold. The mean bias was calculated as the mean difference between the predicted and observed values of tall tree density across all transects:

$$\text{Mean bias}=\frac{1}{n}\sum_{i=1}^{n}\left({\hat{y}}_{i}-y_{i}\right),$$

where ${\hat{y}}_{i}$ is the predicted value, and $y_{i}$ is the observed value for transect *i*, and *n* is the total number of transects (*n* = 900). This metric provides a straightforward indication of whether the model tends to systematically overestimate or underestimate tall tree density.

After cross‐validation, the final model is fitted using the entire available dataset (all 900 transects) and is used to predict giant tree density across the study area, considering environmental factors as predictor variables. The final RF model refers to the structure (i.e. hyperparameters and predictor variables) that achieved the best performance during spatial cross‐validation. The importance of environmental variables was analyzed using marginal plots, holding the other variables constant at a mean value. This approach consisted of a sensitivity analysis in which the importance of variables is measured by permuting variables in the model and measuring the increase or decrease in tree density.

Finally, the parameters of the final RF model were applied to stacked environmental layers at the pixel level across the entire Amazon biome using map algebra, producing spatially explicit density maps for the uppermost strata of the forest canopy, with emphasis on giant trees (≥ 60 m) (Fig. S1). All statistical and spatial modeling and analysis procedures were conducted in the R environment v.4.2.1 (R Core Team, 2024), using the Mass (Ripley *et al*., 2022) and randomForest (Cutler & Wiener, 2022) packages.

### Assessing biomass estimates

To estimate AGB as a function of tall tree density in the Amazon biome, we used LiDAR transect biomass reference data developed by Ometto *et al*. (2023). To ensure consistency and methodological independence from interpolated values, we recalculated AGB per transect based on observed biomass estimates. Each 3.75 km^2^ transect was subdivided into square pixels, and biomass was estimated per pixel using LiDAR‐derived structural metrics calibrated with field inventory data. These pixel‐level values were then aggregated using the PRODES forest mask (250 m × 250 m resolution), retaining only those pixels classified as forest. For each transect, total AGB was calculated by summing the biomass values of all intersecting PRODES‐classified pixels. Finally, AGB values were converted from megagrams per hectare (Mg ha^−1^) to gigatons per square kilometer (Gt km^−2^), enabling a standardized interpretation of biomass stocks at broader spatial scales. This harmonization of spatial resolution and biomass estimation ensured coherence between the tall tree density and AGB datasets while minimizing redundancy in data derivation.

To assess the relationship between tall tree density and biomass stocks across the Amazon biome, we initially fit a linear model (AGB ~ density_of_tall_trees) to quantify the strength and direction of the correlation, reporting the coefficient of determination and significance level. However, for better visual interpretation and to account for nonlinear trends – especially at higher density levels – a locally weighted smoothing function (LOESS) was applied to the scatterplot. This methodological approach enables a robust analysis of the role of tall trees in biomass stocks in the Amazon, contributing to the understanding of the spatial distribution of carbon stored in tropical forests and the factors that influence this dynamic at different geographic scales.

## Results

### Density of tall trees and underlying environmental factors

LiDAR mapping detected 5522 736 trees, with an approximate average of 767 trees km^−2^ in *c*. 7.2 km^2^ of overflown area. The maximum height found was 88.5 m, validating this information in the field as the tallest known tree in South America (Gorgens *et al*., 2019). The density of tall trees showed weak to moderate positive correlations with the FAPAR (*R* = 0.13), the number of clear days (*R* = 0.11), and soil clay content (*R* = 0.12), while displaying negative associations with wind speed (*R* = −0.27), precipitation seasonality (*R* = −0.02), and lightning frequency (*R* = −0.34), indicating potential limiting effects of wind and disturbance on the occurrence of giant trees (Fig. S3). The observed density of trees ≥ 60 m per transect ranged from 0 to 227 trees km^−2^ (see Dataset S1; Fig. S4a,b). The correlation structure revealed moderate to strong associations among several climatic variables. Variables, tannual, tmax, and tseason were strongly positively correlated (*R* > 0.7), as were pannual and pwettest. These patterns reflect expected coherence between related environmental drivers (e.g. different dimensions of temperature or precipitation). Conversely, wind variables (uspeed and vspeed) showed weak to moderate correlations with most other predictors.

**Fig. 2:**
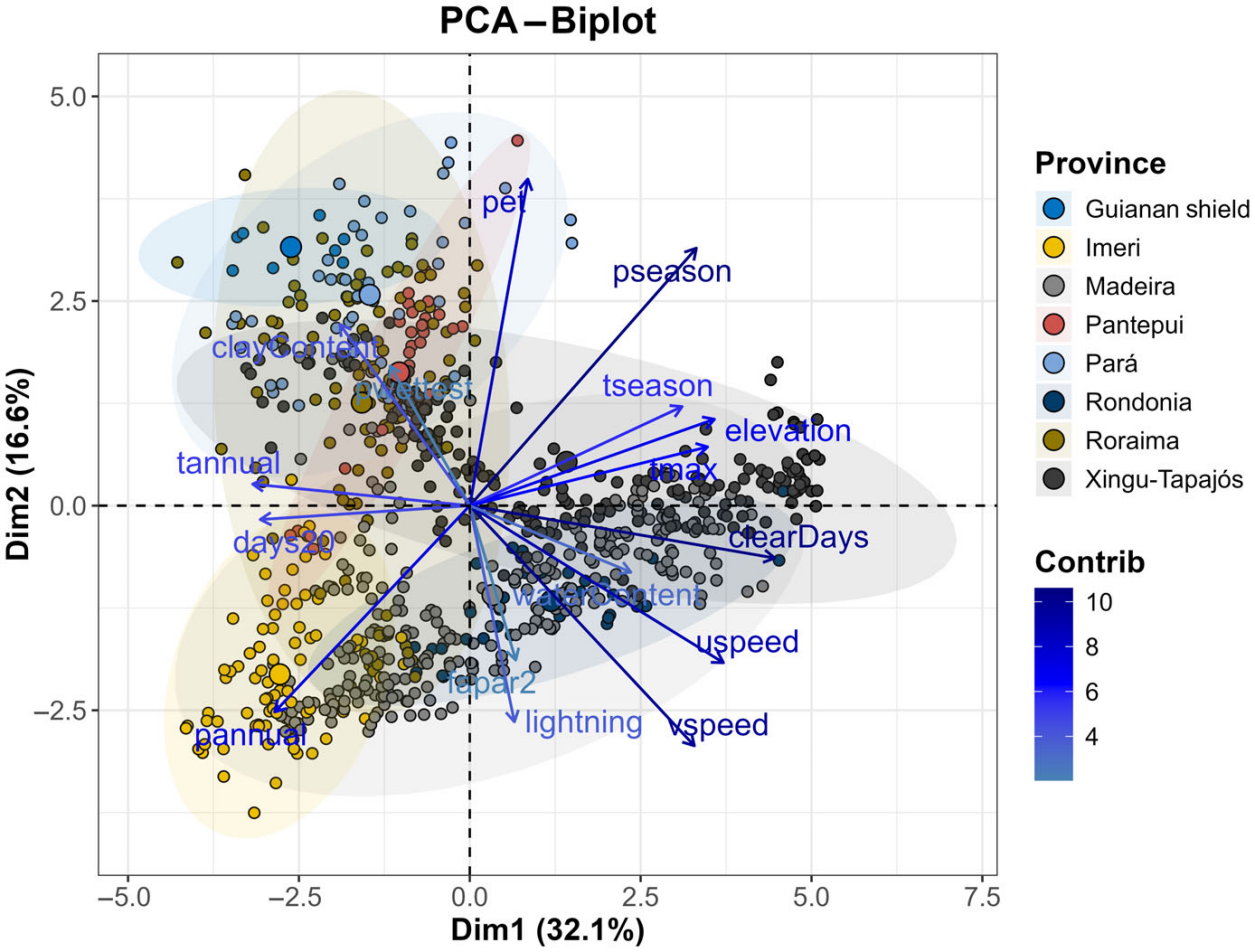
The biplot shows the first two principal components of the principal component analysis (PCA), together explaining 48.7% of the data variance. The length of the arrows indicates the degree of contribution (influence) of the environmental variables to the principal axes. Variables with longer arrows (such as pseason, pet, and lightning) significantly impact discriminating biogeographic provinces. The direction of the arrows indicates correlation: vectors that are close or aligned indicate positive correlation. Vectors that are opposite (> 90°) indicate a negative correlation. For example, pseason positively correlates with Dim1, whereas uspeed and vspeed correlate negatively. The arrow colors reflect each variable's relative contribution to the model, with a gradient from light blue (low contribution) to dark blue (high contribution). Variables with high contribution best explain the observed variation between provinces, such as pet and lightning. Each point represents a sampling unit (site) within a specific biogeographic province. Point sizes in the biplot are scaled using a transformation (1 + tree density) to enhance visual clarity while retaining zero‐density information. Ellipses group provinces based on their environmental similarities. Example: Guiana Shield (blue) is associated with specific environmental features such as elevation. Pantepui (red) is strongly associated with clayContent and waterContent. The size of the dots reflects the density of tall trees (contribution of giant species) in each biogeographic province. Areas with larger dots indicate a higher density of tall trees, possibly associated with variables such as lightning and clearDays.

PCA revealed the dominant environmental gradients across the Amazon (Fig. 2) and within provinces. The first two components explained 48.7% of the variance (PC1 = 32.1%, PC2 = 16.6%). PC1 summarized broad climatic–disturbance contrasts and water balance, whereas PC2 captured edaphic/topographic variation. These axes separated provinces along moisture–disturbance and soil–terrain gradients, respectively; variable loadings and biplots are shown in Fig. 2.

PC1 was primarily structured by climatic variables, including precipitation seasonality (pseason) and potential evapotranspiration (pet), whereas PC2 reflected a combination of disturbance‐ and soil‐related factors, including wind speeds (uspeed, vspeed), clay content, and lightning frequency. The spread of data points in PCA space showed partial separation of biogeographic provinces, indicating regional differences in environmental conditions.

While PCA does not predict tall tree density directly, the ordination helps contextualize environmental heterogeneity. For instance, provinces such as Guiana Shield and Roraima were positioned in regions of PCA space associated with lower wind speed, higher clay content, and fewer clear days – conditions that may indirectly favor the persistence of tall trees. By contrast, provinces like Pará and Pantepui clustered in areas associated with stronger wind regimes, higher climatic seasonality, or more edaphically constrained conditions, aligning with their generally lower density of giant trees (see also Fig. S2). Province‐level PCAs further revealed differences in the correlation structure of environmental variables. These local‐scale analyses complement the biome‐wide PCA by illustrating how the relative influence and collinearity of predictors may vary across ecological regions, supporting the interpretation of region‐specific environmental constraints on tall tree distributions.

### Spatial modeling

Our analysis confirmed, with a high level of accuracy, that the fitted RF model (*k*‐fold cross‐validation: RMSE = 4.8 trees km^−2^) indicates a general spatial trend in the density of tall trees throughout the Amazon basin. The spatial model explained 79% of the variation in tall tree density and presented a significant correlation between the observed and estimated values (*R* = 0.96; RMSE = 10.45, Fig. S4a). The distribution of predictions closely matched observed values (Fig. S4b), although the model tended to slightly underestimate higher densities. Residual analysis revealed systematic overestimation at low densities and underestimation at higher values, indicating some nonlinearity not fully captured by the model.

The results from the spatial cross‐validation demonstrated satisfactory performance of the RF model in estimating the density of tall trees. The RMSE was 10.5 trees km^−2^, indicating reasonable accuracy in the predictions. By contrast, the mean coefficient of determination (*R* ^2^) of 0.6 shows that the model could explain a substantial portion of the variability in the observed data. Additionally, the mean bias of 0.4 reveals a low systematic deviation, suggesting that the model showed good overall accuracy without tendencies toward underestimation or overestimation. Spatially, most transects exhibited low residuals, but localized deviations in northeastern Amazonia suggest areas with greater environmental heterogeneity or reduced predictor performance (Fig. 4c,d). Transects with exceptionally high‐observed densities of giant trees tended to have their values underestimated by the model. This pattern likely reflects the rarity of such high‐density transects in the training data and highlights a common limitation of machine learning models in capturing extreme values.

**Fig. 3:**
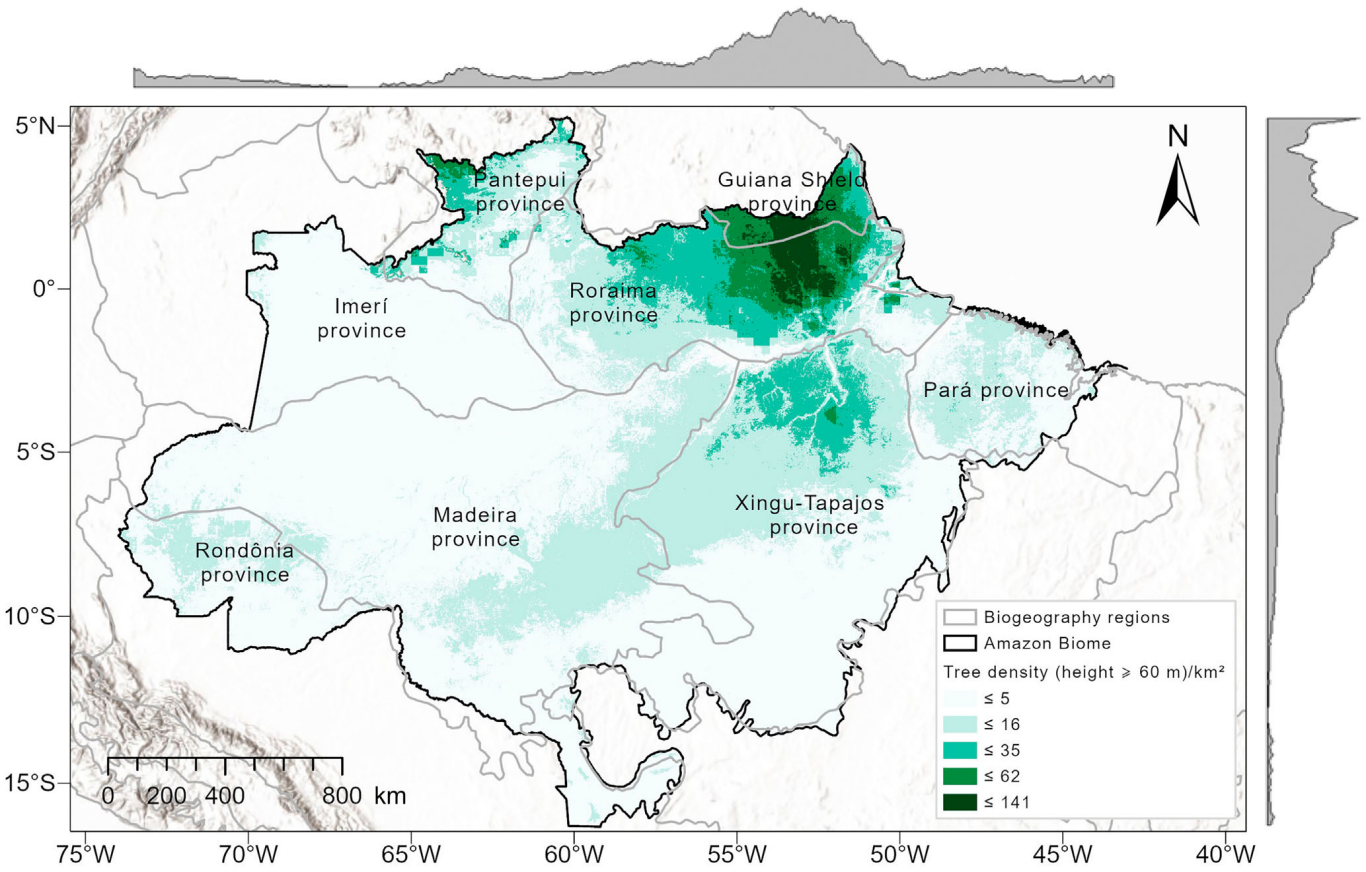
Potential map generated by the random forest spatial model to define ideal zones for the occurrence of high density of giant trees in the Amazon. The areas are color coded according to different density ranges, where lighter shades indicate lower tree density (≤ 5 trees km^−2^), darker shades indicate higher density (up to 141 trees km^−2^). Gray lines delimit major biogeographic provinces within the Amazon biome (such as the Guiana Shield, Xingu‐Tapajós, and Roraima), showing distinct density patterns across the Amazon region. Gray shading on the top and side of the map represents latitudinal and longitudinal distribution of density. The highest densities of giant trees are concentrated in the Guiana Shield province and northern Roraima province. By contrast, potential areas with near‐zero density of giant trees include parts of the Madeira and Rondônia provinces as well as extensive regions in the southwestern Amazon. The map is available at https://doi.org/10.5281/zenodo.13850976.

**Fig. 4:**
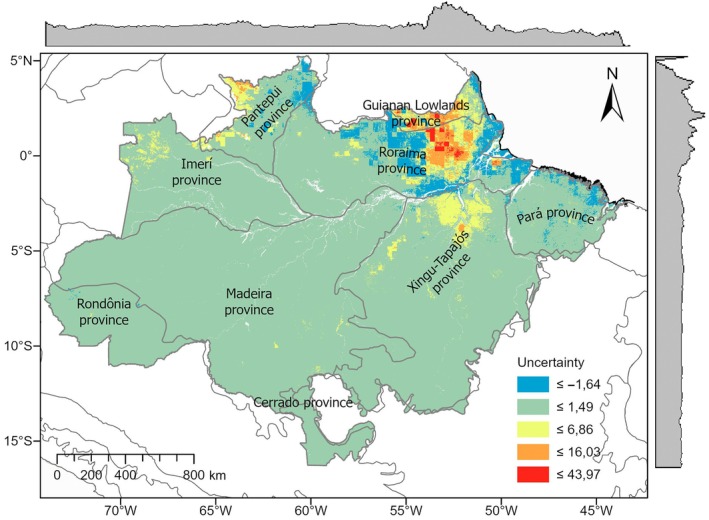
Relative prediction uncertainty for tall‐tree density (height ≥ 60 m) across the Brazilian Amazon. Uncertainty is expressed as the standardized width of the prediction interval (*z* score of pixel‐level PI width from spatial cross‐validation/predictive distribution). Thus, negative values indicate below‐average uncertainty, and positive values indicate above‐average uncertainty. Legend colors denote nonoverlapping value ranges (classes), with warmer colors indicating higher uncertainty and cooler colors indicating lower uncertainty. Biogeographic provinces are delineated for reference. Uncertainty is generally low across most of the biome but is concentrated in the northeast, particularly in the Guianan Lowlands, Pará, and Roraima provinces. Gray marginal bars at the top and right depict the latitudinal and longitudinal distributions of modeled tall‐tree density.

The spatial model prediction of large tree density shows differences among biogeographic provinces, Fig. 3, with markedly higher values in the northeastern portion of the Amazon, where there is a predominance of *terra firme* forests in the northern Guiana Shield and the eastern portion of the Roraima biogeographic provinces (darker area on the map, *c*. −58 to −51° longitude, and −4° to −3° latitude). The density of tall trees in these areas may exceed 120 individuals km^−2^, estimated by the spatial model (Figs S4b, 3). Note that these regions are characterized by a low incidence of lightning and lower wind speeds (Fig. S2). Densities close to zero can be seen in large portions of the south‐central eastern Amazon (Pará, Xingu‐Tapajós, and Madeira provinces; note that these biogeographic provinces coincide with the arc of deforestation). We also noted lower densities throughout the Imerí province and the entire northern portion of the Madeira province. This westernmost strip of the biome is characterized by the solid influence of the seasonality of the dry season (Fig. S2). Most regions, including the Madeira, Rondônia, and Xingu‐Tapajós provinces, exhibited low to moderate uncertainty (≤ 6.86 trees km^−2^), reflecting well‐sampled areas with more predictable environmental conditions. By contrast, higher uncertainty (> 16.03 trees km^−2^) was concentrated in northeastern Amazonia, particularly in parts of the Guianan Lowlands, Pará, and Roraima provinces (Fig. 4). Prediction uncertainty is heteroscedastic: absolute errors and interval widths increase with the predicted mean density, particularly in the highest‐density bins, whereas relative errors remain comparable across most of the range. This scale effect is as important as variation in sampling intensity or environmental predictability (Fig. S4d).

Across the 4.2 million km^2^ Brazilian Amazon biome, the spatial model estimates *c*. 55.5 million trees taller than 60 m (0.0001% of the estimated ≈ 390 billion trees for the Brazilian Amazon biome), with an average density of 13.5 (±4.5) tall trees km^−2^. However, we found a sizeable spatial aggregation of these tall trees, with *c*. 14% of tall tree density occurring in 1.28% of the total area of the Amazon (latitudes −2° to 3° S and 53° to 58° W) and 50% occurring in 11.2% (latitudes −5° to 5° S and 51° to 60° W).

The essential variables according to %IncMSE for the model were pannual (13.01%), tannual (11.89%), uspeed (11.65%), and clayContent (10.98%) (Fig. S5b). Meridional wind speed (N–S, m s^−1^) (vspeed) and lightning rate, claycontent, and number of clear days per year (clearDays) were found to be the primary environmental factors associated with decreases in spatial model residual variance (IncNodePurity) and contributed significantly to separating the observations into more homogeneous groups along the trees in the RF model (Fig. S5a). High mean and maximum temperatures also negatively affected the density of tall trees. Tree density increased with photosynthetically active radiation (fapar) by > 70% but decreased with the number of clear days linked to direct radiation (Fig. S6). Mean annual precipitation (pannual) and precipitation regimes (pwettest, days20), as well as soil clay content (Claycontet), elevation above sea level (m) (elev), and temperature seasonality (tseason), indicate increases and stability in spatial model‐estimated density values. Although water availability is a fundamental driver of tall tree density, we observed that biome‐wide relationships between climate‐based indicators – such as precipitation seasonality (pseason) and potential evapotranspiration (pet) – and tall tree density were generally weak or inconsistent. This suggests that large‐scale climatic seasonality alone does not fully explain the spatial variation in giant tree occurrence. One possible explanation is that local‐scale ecological processes, including topographic heterogeneity, soil buffering capacity, or historical disturbance regimes, may mask or override broader climatic signals in shaping density patterns (Fig. S7). By contrast, the effect of soil water content showed a clearer pattern: a generally positive or unimodal response, where tall tree density declined under very low water content (indicative of stress), but increased once a certain threshold of soil moisture was surpassed (Fig. S6).

### Implications for biomass estimates

**Fig. 5:**
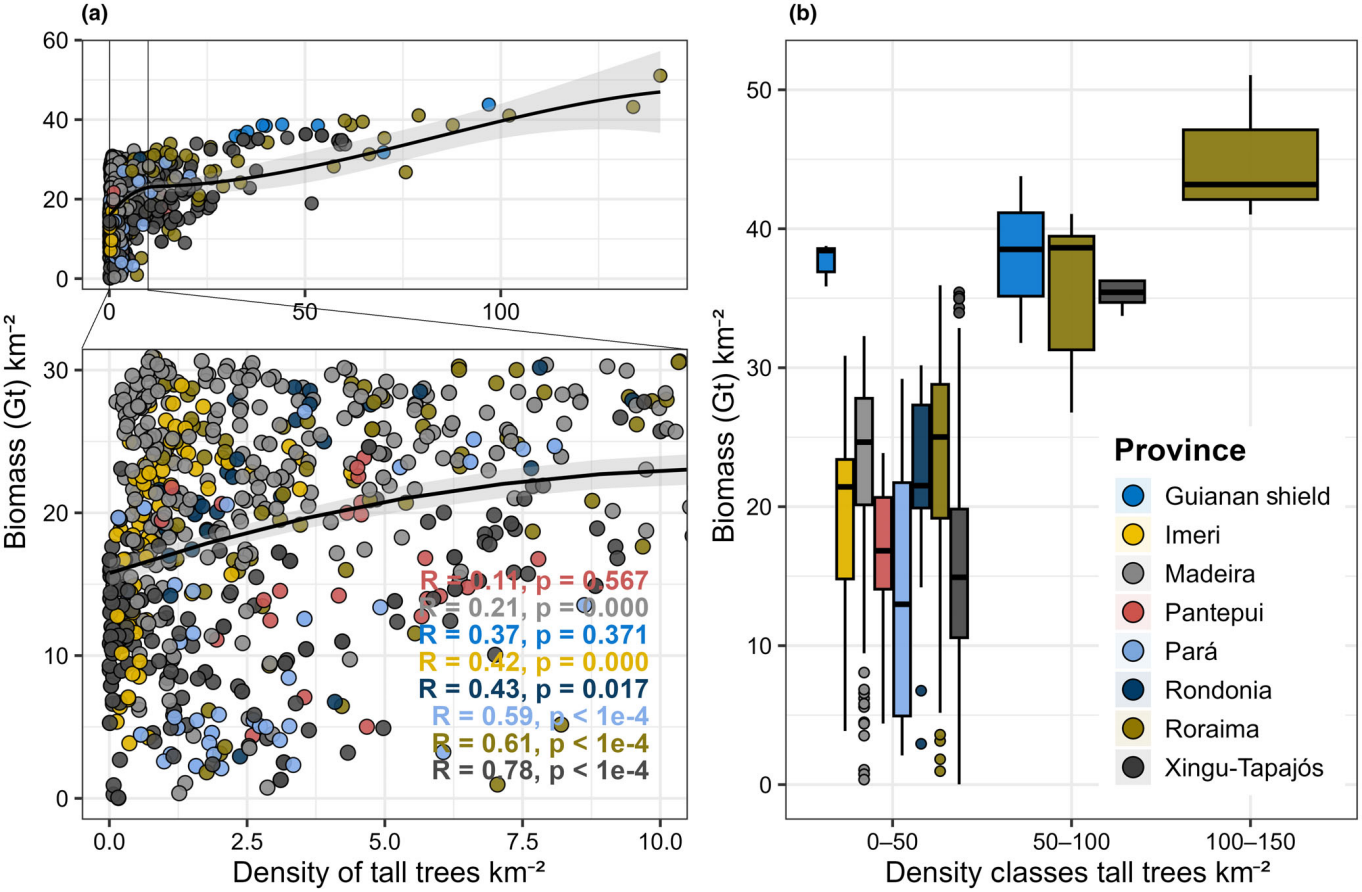
Relationship between aboveground biomass (AGB) and the density of tall trees (i.e. ≥ 60 m) for the 900 LiDAR plots studied in the Amazon biome. (a) Scatterplots colored by biogeographic province, showing AGB (Gt km^−2^) as a function of tall tree density (trees km^−2^). The black curve is a LOESS smoother to aid visual interpretation. The inset colored annotations report province‐specific linear model statistics (*R* and *P*) used to quantify association strength; linear regression lines are not plotted to avoid over‐plotting and misleading linearity when the relationship is non‐linear at higher densities (gray shading representing confidence intervals (CI = 95%)). The top panel highlights density values <10 trees km^−2^, while the bottom panel shows the full distribution. (b) Boxplots of biomass values across three tall‐tree density classes (0–50, 50–100, and 100–150 trees km^−2^). The horizontal lines in the boxplots represent the first, second (median), and third quartiles of the biomass data distribution. The vertical lines indicate the interquartile range of the data.

At a regional and biogeographic scale, we compared our tall tree density estimates with the potential AGB stock map produced by the same airborne LiDAR campaign as this study (Ometto *et al*., 2023). As expected, a higher density of tall trees is strongly associated with higher AGB (*R* = 0.5, *P* < 2.2e−16) (Fig. 5a). These relationships are more pronounced where large tree density is higher, specifically in the provinces of Roraima and Guiana Shield, which also correspond to the largest AGB densities across the biome. Interestingly, Xingu‐Tapajós and Madeira have relatively high AGB even in areas with a low density of tall trees.

However, in areas with higher densities of tall trees, the relationship is not linear and may indicate saturation at extreme density rates. For example, areas with low density of tall trees may still have significant biomass levels due to the contribution of smaller trees. The relationship between giant tree density and AGB was also analyzed in different classes of tree density (0–50, 50–100, and 100–150 trees km^−2^) (Fig. 5b). We observed that, across the biome, spatial patterns agree well and that our density estimates are predictive of biomass even in biogeographic provinces with densities of up to 50 tall trees km^−2^. Notably, the relationship between biomass and density is most significant in areas with > 100 tall trees km^−2^ (Fig. 5b). The results reveal a consistent increase in biomass with increasing tall tree density, mainly in Roraima and the Guiana Shield. The classes with the highest density (100–150 trees km^−2^) presented the highest median biomass values (40.0–50.0 Gt km^−2^) and the lowest variability, while the lower density classes (0–50 and 50–100 trees km^−2^) exhibited more visible dispersion in biomass values, reflecting structural heterogeneity.

## Discussion

### Patterns of tall tree density and environmental drivers

The density of tall trees is a valuable input for modeling large‐scale biological and biogeochemical processes and a prominent component of ecosystem structure, governing elementary processes such as carbon, water, and nutrient retention, competitive dynamics, and habitat suitability for many plant and animal species (Slik *et al*., 2013; Bastin *et al*., 2018; Pinho *et al*., 2020). Our results show that a complex combination of environmental factors influences the density of tall trees in the Amazon. Clay‐rich soils, moderate precipitation and temperature, and low incidence of lightning and strong winds create stable conditions for a high density of tall trees. These findings are consistent with the existing literature highlighting the importance of water availability and stable climatic conditions for the growth and maintenance of large trees (Venter *et al*., 2017; Gorgens *et al*., 2021). Areas located in the northeastern portion of the biome, between the provinces of Roraima and the Guiana Shield (*c*. −58° to −51° longitude, and −4° to −3° latitude), have the highest local density of tall trees across the Amazon. We estimate up to 140 individuals per km^2^ in these areas, where well‐drained hilltops *terra firme* forests predominate. These patterns confirm previous results at the same scale for biomass (Ometto *et al*., 2023), maximum height (Gorgens *et al*., 2021), and diversity of large species (De Lima *et al*., 2023). Furthermore, these areas are among the most remote and least explored in the Amazon (Carvalho *et al*., 2023), suggesting a possible role of a low current human population density and the absence of roads or large infrastructures as factors controlling the occurrence of tall trees. Being isolated areas with low anthropic pressure enhances the conservation value of these areas.

Lightning and strong winds associated with convective storms and cold fronts in the southern and western Amazon can cause significant mortality of tall trees through damage, toppling, or structural failure (Arellano *et al*., 2019; Cushman *et al*., 2021; Ibanez *et al*., 2024). The relative height and canopy exposure of giant trees makes them particularly vulnerable to these disturbances (Rifai *et al*., 2016; Cushman *et al*., 2021; Ibanez *et al*., 2024). In regions where tall trees are scarce, this may reflect selective mortality pressure exerted by recurrent windthrow or lightning events, rather than an absence of disturbance. Such filtering processes may help explain regional variations in tall tree density across the biome. Recurrent increases in wind speed can significantly modify density patterns in many regions (Marra *et al*., 2014). Wind and lightning act as disturbance constraints across tropical forests of varying canopy height. While exposure and crown architecture likely amplify risk for tall trees (≥ 60 m), empirical studies demonstrate that lightning is a leading cause of large‐tree mortality even in forests with 25–45 m canopies (Gora *et al*., 2020; Yanoviak *et al*., 2020; Gora & Esquivel‐Muelbert, 2021). Since most trees in the biome are not tall (average canopy height 30–45 m, e.g. Lang *et al*., 2023), lightning and wind are much less critical for forests with lower canopies (Zoletto *et al*., 2023). In our maps, provinces with higher wind speed and lightning frequency show lower modeled densities of giant trees, consistent with these agents as limiting factors rather than implying they are unimportant in shorter forests.

At local scales, topography and hydrological conditions play a critical role in shaping the density of tall trees. Our results support previous findings that giant trees are more frequently associated with elevated terrains, such as plateaus, which offer more stable edaphic and hydrological conditions (Jucker *et al*., 2018; Zuleta *et al*., 2020). By contrast, lowland areas, particularly those < 50 m above sea level and close to river systems, experience frequent or seasonal flooding that imposes oxygen and nutrient limitations on root systems (Meinzer *et al*., 2001; Koch *et al*., 2004). These conditions limit vertical growth by favoring species adapted to flooding stress over those that invest in structural height (Schöngart *et al*., 2002; Durgante *et al*., 2023). Moreover, hydrological variability and sediment loads can lead to unstable nutrient availability, further constraining the development of tall trees (Koch *et al*., 2004; Bittencourt *et al*., 2020; Bartholomew *et al*., 2022). Because tall trees have high energy and hydraulic demands, particularly for transporting water and nutrients over long vertical distances, any restriction in root oxygenation can compromise their growth and survival (Domec *et al*., 2008; Couvreur *et al*., 2018; Skiadaresis *et al*., 2021). Thus, lowland and seasonally flooded environments tend to support tree communities with shorter statures, better suited to mechanical and physiological stability under these challenging conditions.

Our findings also indicate that clay and soil water content are critical for the occurrence and density of giant trees in the Amazon, as they directly influence nutrient availability and water stability, which are essential for the growth of these trees (Quesada *et al*., 2011; Spanner *et al*., 2022). Soils with high clay content have a higher cation exchange capacity, which allows the retention of essential nutrients such as calcium, magnesium, and potassium, favoring the sustained growth of trees. In addition, clay contributes to the formation of aggregates in the soil, improving its structure and increasing water retention. In Amazonian regions with clayey soils, giant trees' density and biomass are higher than in areas with sandy soils due to their water retention capacity (Durgante *et al*., 2023). Soil water availability, especially during dry periods, is crucial for the survival of these trees, and clayey soils provide a stable source of moisture. However, other studies suggest that in the tropics, higher soil fertility is often associated with lower maximum canopy height (Muller‐Landau *et al*., 2021; Jucker & Ali, 2023). One proposed explanation is that faster‐growing plants in fertile soils may prioritize growth over structural defenses or longevity, resulting in increased vulnerability to disturbances such as windthrow (Jackson *et al*., 2021; Joswig *et al*., 2022; Durgante *et al*., 2023; Sanchez‐Martinez *et al*., 2025). While this growth–defense trade‐off hypothesis offers a compelling framework, especially in explaining species‐level differences in wood density and mortality risk, it remains challenging to test directly and should be interpreted as a conceptual model in need of further empirical validation.

In environments well drained with regular rainfall and defined seasons, there is a constant supply of water in the soil, which is essential for continued growth and for the maintenance of complex physiological processes that require high levels of water, such as transpiration and photosynthesis for many tall trees (Fig. S5b), and this is directly related to the number of days with clouds or days with few clouds (‘clear days’). An increase in cloudless days indicates a greater incidence of sunlight and high temperatures, which can increase the rate of photosynthesis, favoring tree growth to a thermal tolerance threshold (Green *et al*., 2020; Sullivan *et al*., 2020).

Although atmospheric drought – driven by increased temperatures, clear‐sky radiation, and reduced precipitation – can temporarily enhance photosynthetic activity when water is abundant (Aguirre‐Gutiérrez *et al*., 2019; Green *et al*., 2020), prolonged drought conditions increase the risk of hydraulic failure, particularly in tall trees. Taller individuals are thought to be more vulnerable to xylem embolism due to longer water transport pathways and higher evaporative demand under exposure to intense solar radiation (Oliveira *et al*., 2019; Garcia *et al*., 2023; Mattos *et al*., 2023). Indeed, Bennett *et al*. (2015) found a consistent negative effect of drought on the growth and survival of large trees at the global scale. However, it is important to note that this pattern is not universal–studies such as (Fauset *et al*., 2012; Zuleta *et al*., 2017) have shown that smaller trees can also exhibit elevated mortality under drought conditions, depending on forest structure, rooting depth, and competition for light and water. Therefore, while tree height is a key factor influencing drought vulnerability, drought‐induced mortality patterns are context‐dependent, and shaped by the interaction of multiple structural and environmental factors. On broader scales, climatic drivers such as the number of clear days and precipitation seasonality have also been linked to spatial patterns in tree density (Bruijnzeel *et al*., 2011; Wagner *et al*., 2016; Jiang *et al*., 2017; Ehbrecht *et al*., 2021).

### Importance of giant trees density for biomass stocks

The positive relationship between tall tree density and biomass for the entire biome and biogeographic provinces highlights the importance of giant trees, which play an essential role in biogeochemical processes despite representing a small fraction of the total trees (Bastin *et al*., 2018; Ali & Wang, 2021). For example, in the tropics, Sullivan *et al*. (2020) showed that only 1% of trees in tropical forests account for *c*. 50% of AGB. Slik *et al*. (2009, 2013) suggest that the impact of large trees (dbh ≥ 70 cm) on AGB is, on average, 25.1% in South America and more than two‐thirds at the pantropical scale. These results are consistent with the meta‐analysis by de Lima *et al*. (2022) in the Amazon biome, which highlighted that only one giant tree (dbh ≥ 70 cm) could accumulate 82% of all stored AGB Mg ha^−1^. In general, tall tree density maps can potentially characterize habitat heterogeneity directly (Tuanmu & Jetz, 2015; Bastin *et al*., 2018), so canopy height has been classified as a high‐priority biodiversity variable to be observed from space (Lang *et al*., 2023). Therefore, the density of tall trees can be used as a direct indicator of the potential for carbon storage in tropical forests.

Our comparison between giant‐tree density and LiDAR‐derived AGB should be interpreted as a relationship between two structural metrics derived from canopy height, rather than as an independent validation. The AGB surface is modeled from top‐canopy height calibrated with field plots (Ometto *et al*., 2023), whereas giant‐tree density reflects the upper‐tail exceedance of the height distribution (height ≥ 60 m). This shared dependence on LiDAR height introduces partial circularity, making a positive association unsurprising. We therefore avoid causal language and frame the result as co‐variation within canopy structure: AGB largely captures stand‐level central tendency of height and biomass, while giant‐tree density captures the frequency of extreme canopy elements. Future work will quantify the incremental information provided by the exceedance metric (e.g. partial correlations or SEM controlling for mean or P90 height) and, where possible, confront both layers with fully independent field‐based biomass estimates.

Our study did not test the effect of local processes influencing the density of tall trees on AGB. However, a possible explanation for the high AGB in biogeographic provinces dominated by large trees may be related to the reproductive strategy of wind dispersal of some species, that is they need to be tall and emergent to effectively disperse their seeds and exceptionally maximize their geographic influence (Slik *et al*., 2013, 2024). In the Amazon, the most abundant larger species (dbh ≥ 70 cm) were *Goupia glabra*, *D. excelsa*, *Aspidosperma excelsum*, *Couratari guianensis*, *Manilkara huberi*, *Dipteryx odorata*, *Tabebuia serratifolia*, *Bertholletia excelsa*, and *Caryocar villosum* (de Lima *et al*., 2022). These species represented *c*. 20% of the individuals sampled in 240 plots inventoried in the eight biogeographic provinces in the biome. Therefore, although environmental controls influence maximum tree height and the diversity of large species at a regional scale (Gorgens *et al*., 2021; De Lima *et al*., 2023), this dominance is also driven by species‐specific traits that provide the ability to reproduce and support large biomass stocks at a local scale (Loubota Panzou *et al*., 2018). Different strategies in seed dispersal are linked to maximum tree height and aggregation patterns (Wunderle, 1997; Clark *et al*., 2005; Slik *et al*., 2024). In particular, wind‐dispersed species such as *D. excelsa*, *A. excelsum*, and *T. serratifolia* show more aggregated patterns than animal‐dispersed species such as *B. excelsa* and *M. huberi*, which may affect large tree density and AGB at the local scale, as animals are more effective at dispersing seeds in a more uniform spatial distribution (Wright *et al*., 2003). This finding emphasizes the importance of including species traits and changes in species composition as explanations for AGB gradients in tropical forests (Slik *et al*., 2009, 2013; Loubota Panzou *et al*., 2018).

Another critical factor at the local scale is gap formation, which provides opportunities for seed germination and recruitment, especially in areas where light is a limiting resource. Higher densities of tall trees produce more significant gaps when they fall (Arasa‐Gisbert *et al*., 2021; Reis *et al*., 2022), although the frequency and size of these gaps are also influenced by disturbances such as wind and lightning (Hubbell *et al*., 1999; Cramer *et al*., 2007; Cushman *et al*., 2021). A relatively low proportion of significant gaps is observed in the provinces of Roraima and Guiana Shield, where we found the highest densities of tall trees and stored biomass (probably caused by the low mortality rate of large trees caused by strong winds and lightning). These results suggest that low gaps may limit the recruitment of tall trees, contributing to their aggregated patterns and more prominent AGB stock over time, as germination and recruitment may be limited to fewer areas with more significant gaps. For example, clusters of *D. excelsa* and *T. serratifolia* can be found in an aggregated form, likely due to a combination of anemochoric seed dispersal, a potential dependence on light for germination and seed recruitment, as well as local topography (lowlands vs plateaus) that favor their aggregation patterns (Lewis *et al*., 2017; Ali *et al*., 2020; de Lima *et al*., 2022).

The results presented here reinforce the hypothesis that preserving areas with a high density of giant trees is crucial to maintaining ecosystem services associated with carbon storage. Including tall tree density as a critical variable in biomass estimation models can substantially improve the accuracy of these estimates. These models need to better account for the role of these large trees, and airborne LiDAR surveys are practical tools to detect and quantify large trees and quantify their contributions to biomass stocks (Asner *et al*., 2010; Coomes *et al*., 2017). This is especially interesting in difficult‐to‐access regions with limited ground‐based measurements. The observed relationship suggests that the loss of tall trees due to deforestation or forest degradation can significantly reduce biomass stocks. Inadequate management that does not consider the conservation of these trees can have drastic implications for the carbon budget, amplifying the impacts of glasshouse gas emissions.

### Broader implications and concluding observations

The main findings of this study are significant for understanding the ecology and conservation of giant trees in the Amazon biome. We also detail some of the main limitations of the study that can be seen in notes S1. Mapping tall tree density provides valuable insights into forest structure and health, which are crucial for biodiversity conservation and climate change mitigation. The data generated can inform forest management and conservation strategies, helping to identify areas in need of protection or restoration. Furthermore, by linking tree density to environmental variables, the study can reveal the key factors influencing the distribution and health of tall trees and offers a beacon of hope in the face of climate change and deforestation, providing a roadmap for more effective conservation efforts. Practical application of the data must be accompanied by a deep understanding of ecological interactions and an adaptive approach to environmental management.

The relationship between tall tree density and local factors, such as soil characteristics, dispersal strategies, and gap formation, is a promising area for future investigation. The hypothesis that local density correlates with reproductive success and recruitment can be tested in different soil types and terrains. Furthermore, exploring density patterns across topographic gradients and relating them to climatic and edaphic variables can provide insights into the interaction between regional and local factors. Regional factors, such as wind and lightning, shape tall tree density patterns in the Amazon. However, they are refined at local scales by processes such as dispersal, topography, and gap formation. These results highlight the importance of integrating multiple scales of analysis to understand the ecology of giant trees in the Amazon and provide support for their conservation under a climate change scenario.

This study contributes to the discussion on the relevance of approaches based on high‐resolution data, such as those provided by LiDAR sensors, to better understand tropical forests' structural dynamics. Furthermore, combining tall tree density data with climate change projections could provide insights into the future of carbon storage in the Amazon. Considering their disproportionate importance in biomass stocks, conserving tall trees should be a priority in global conservation initiatives such as REDD+ (Reducing Emissions from Deforestation and Forest Degradation). Investments in remote monitoring and forest inventories that integrate metrics from emerging trees can improve understanding of forest dynamics and support global mitigation strategies.

Although this study does not aim to model the potential impacts of climate change, it is concerning to note that several climate variables strongly associated with giant tree density may undergo significant changes in future climate crisis scenarios. Environmental changes induced by rising temperatures are already being observed. Changes in environmental variables associated with disturbances can significantly negatively impact the density and survival of large trees. For example, the frequency of anomalous events, such as increased storms and lightning strikes, has already been observed (Dale *et al*., 2001; Seidl *et al*., 2017; Bauman *et al*., 2022; Kamimura *et al*., 2022). Furthermore, in the current scenarios projected by the sixth climate report of the Intergovernmental Panel on Climate Change (IPCC), the average global temperature is estimated to increase by 1.5°C between 2030 and 2052. If temperature continues to increase and precipitation levels decrease, drought scenarios in the Amazon will become more frequent, directly impacting all biodiversity (Flores *et al*., 2024). Even in this scenario, only *c*. 15% of the Brazilian Amazon is protected by conservation units, covering only *c*. 58% of the remaining vegetation. Given the ecological importance of giant trees, understanding the effects of climate change on density patterns is critical and it should be explored quickly at finer scales within the Amazon. This knowledge is critical to refining conservation perspectives in a changing world – for example, to what extent are protected areas in the Amazon currently susceptible to impacts induced by climate disruptions, and how can the giant trees withstand or respond to these changes? Efforts to understand how deforestation and climate change interact and mitigate their impacts are urgently needed in light of the high and increasing rates of deforestation in the Brazilian Amazon, which directly threaten sanctuaries of ancient trees throughout the biome.

## Author contributions

RBL designed research; RBL, DAS, MHN, PRLB, PG, JA‐G, CPO, RLCF, JAAS, TJ, DGS, JPO, and EBG performed research; RBL, MHN, TJ, NH, DRAA, JPO, and EBG provided funding; RBL, CPO, and EBG analyzed data; and RBL, MHN, PRLB, JA‐G, CPO, DAS, PG, and EBG wrote the paper, and all authors edited the manuscript.

## Disclaimer

The New Phytologist Foundation remains neutral with regard to jurisdictional claims in maps and in any institutional affiliations.

## Supporting information


**Dataset S1** Complete dataset with the name and metrics of environmental and geographic variables, tree density, and topographic metrics generated by airborne LiDAR in 900 randomly distributed transects in the Brazilian Amazon biome.

**Fig S1** Methodological workflow for mapping giant trees in the Amazon, from LiDAR data collection to Random Forest modeling and spatial density maps.
**Fig. S2** Spatial distribution of 16 environmental predictors across the Brazilian Amazon used in the Random Forest model.
**Fig. S3** Pairwise scatterplots, correlations, and distributions of tall‐tree density and environmental predictors.
**Fig. S4** Model performance and residual diagnostics for tall‐tree density predictions across 900 Amazon transects.
**Fig. S5** Importance of environmental predictors for tall‐tree density estimation based on IncNodePurity and %IncMSE metrics.
**Fig. S6** Partial dependence plots showing the influence of environmental variables on modeled tall‐tree density.
**Fig. S7** Principal component analysis of environmental variables across Amazonian biogeographic provinces.
Please note: Wiley is not responsible for the content or functionality of any Supporting Information supplied by the authors. Any queries (other than missing material) should be directed to the *New Phytologist* Central Office.

## Data Availability

The data that support the findings of this study are availableblank;inblank;Zenodo at https://data.niaid.nih.gov/resources?id=zenodo_4091221. These data were derived from the following resources available in the public domain: https://zenodo.org/records/4091222#.YDe7l2hKjIV.
