# Supplementary material for: Mapping the density of giant trees in the Amazon
Source: New Phytol. 2025 Oct 14;249(1):152–68. doi: 10.1111/nph.70634 (PMC12676077; doi:10.1111/nph.70634)
Supplement: Supplementary file 2 — Fig S1 Methodological workflow for mapping giant trees in the Amazon, from LiDAR data collection to Random Forest modeling and spatial density maps. Fig. S2 Spatial distribution of 16 environmental predictors across the Brazilian Amazon used in the Random Forest model. Fig. S3 Pairwise scatterplots, correlations, and distributions of tall‐tree density and environmental predictors. Fig. S4 Model performance and residual diagnostics for tall‐tree density predictions across 900 Amazon transects. Fig. S5 Importance of environmental predictors for tall‐tree density estimation based on IncNodePurity and %IncMSE metrics. Fig. S6 Partial dependence plots showing the influence of environmental variables on modeled tall‐tree density. Fig. S7 Principal component analysis of environmental variables across Amazonian biogeographic provinces. Please note: Wiley is not responsible for the content or functionality of any Supporting Information supplied by the authors. Any queries (other than missing material) should be directed to the New Phytologist Central Office. [file NPH-249-152-s001.docx]

## New Phytologist Supporting Information

Article title: Mapping the density of giant trees in the Amazon

Authors: Robson Borges de Lima, Diego Armando Silva da Silva, Matheus Henrique Nunes, Paulo R. de Lima Bittencourt, Peter Groenendyk, Cinthia Pereira de Oliveira, Daniela Granato-Souza, Rinaldo L. Caraciolo Ferreira, José A. Aleixo da Silva, Jesus Aguirre-Gutierrez, Toby Jackson, João R. de Matos Filho, Perseu da Silva Aparício, Joselane P. Gomes da Silva, José Júlio de Toledo, Marcelino Carneiro Guedes, Danilo R. Alves de Almeida, Niro Higuchi, Fabien H. Wagner, Jean Pierre Ometto, Eric Bastos Görgens

Article acceptance date: 22 September 2025

The following Supporting Information is available for this article:

**Fig. S1** Methodological flowchart for mapping giant trees in the Amazon, highlighting data collection, processing, and modeling steps. (A) Data collection: airborne LiDAR surveys are employed to identify and record giant trees (height ⩾ 60 m) and location data, ensuring the accuracy of our findings in the Amazon. (B) Data sources and processing: Occurrence data (trees ⩾ 60 m) are meticulously filtered based on latitude, longitude, and tree density per area. (C) Raw data: Comprehensive climate, topographic, and remote sensing databases are used to generate environmental layers and density records of giant trees (D) Final input variables: Data cleaning involves removing outliers, defining the spatial extent, and variable selection for modeling purposes. (E) Spatial tree density modeling by RF model using a climate variable space (e.g., mean annual precipitation and temperature) and other factors. (F) Response curves: Response curves relate the density of giant trees to environmental variables, showing how these variables influence the density. (G) Density maps: Geographic projection of the modeling results, producing maps that show the density of giant trees across the Amazon.


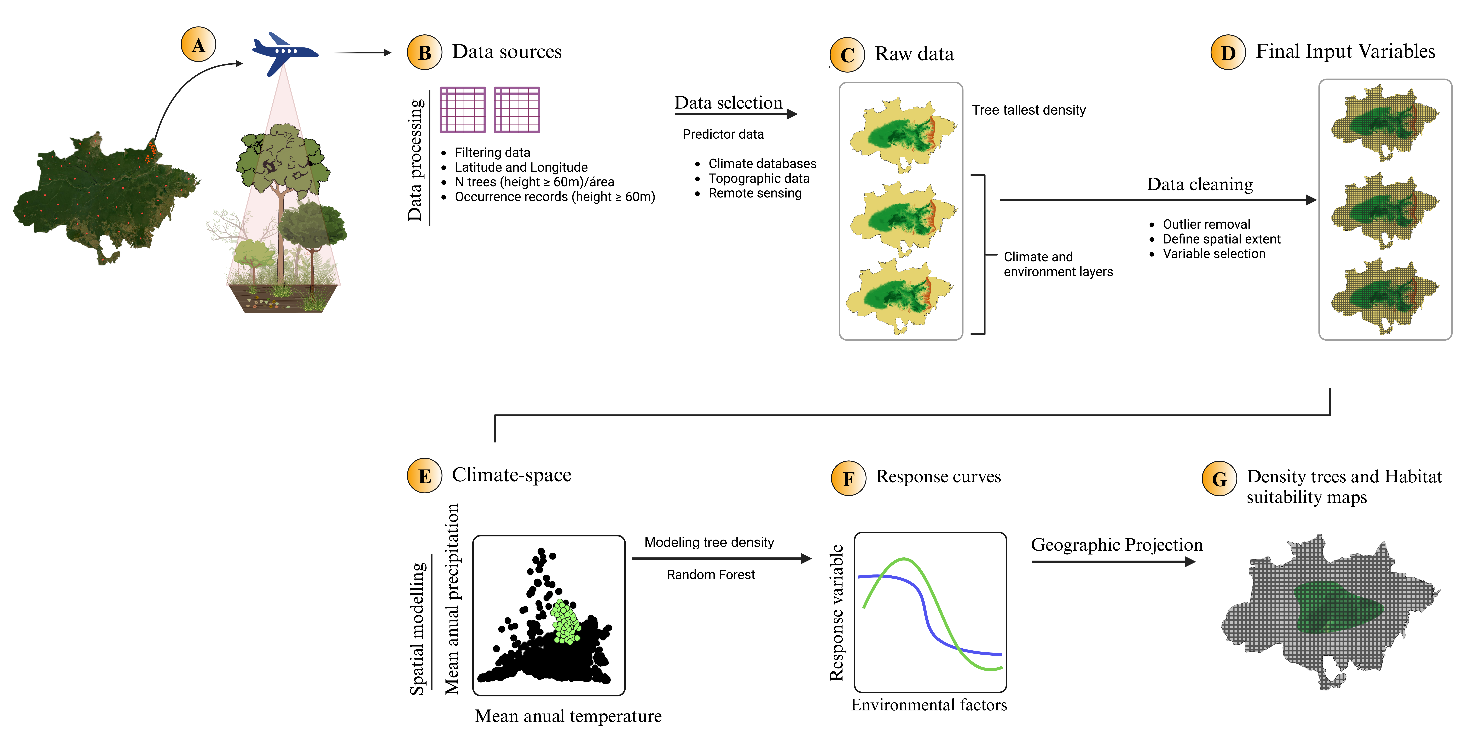


**Fig. S2** Maps of the 16 environmental variables used as predictors distributed in the Brazilian Amazon biome. Each panel represents the spatial distribution of a variable, highlighting its geographic differences. The mapped variables are uspeed and vspeed, wind speed components. clayContent: soil clay content. Lightning: lightning incidence. clearDays: number of clear days throughout the year. pannual: total annual precipitation. Elevation: terrain elevation. pseason and tseason: seasonality of precipitation and temperature, respectively. Pet: Potential evapotranspiration. tmax: average annual maximum temperature. pwettest: precipitation in the wettest month. tannual: average annual temperature. days20: number of days with temperatures above 20°C. fapar2: fraction of photosynthetically active radiation absorbed by plants. waterContent: soil water content. The maps illustrate the spatial variation of each predictor, which is essential for environmental and ecological modeling in the Amazon region.


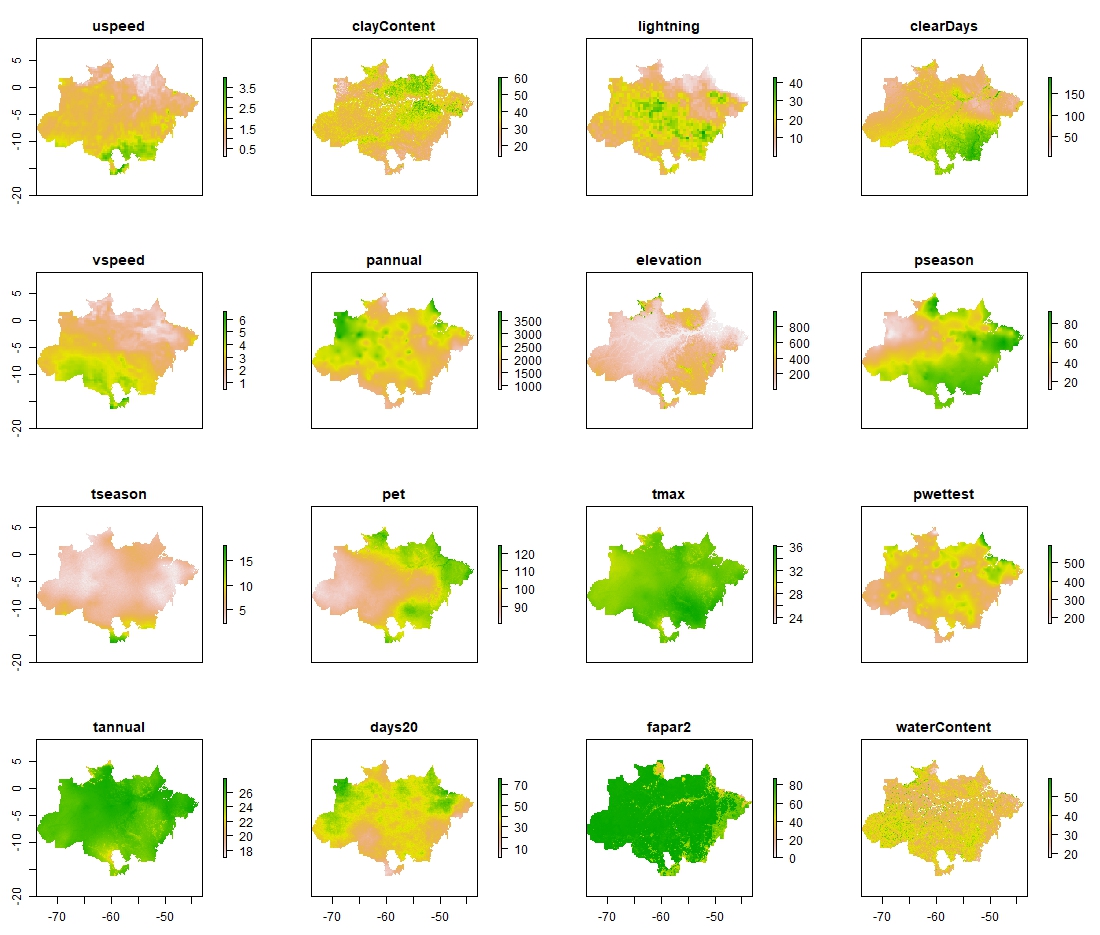


**Fig. S3** Pairwise scatterplots and Pearson correlations among the tall tree density variable and environmental predictors used in the random forest model. Scatterplots in the lower triangle illustrate bivariate relationships, while the upper triangle shows Pearson correlation coefficients (R) for each variable pair. Histograms and density plots on the diagonal represent the distribution of each variable. Variables are shown in natural scale. The density of tall trees per km^2^ (*MDRF_60*) is shown in the first row and column. Strong correlations among related climatic variables (e.g., temperature and precipitation metrics) are evident, as well as distinct relationships between tall tree density and specific predictors such as lightning frequency, wind speed, and clay content.


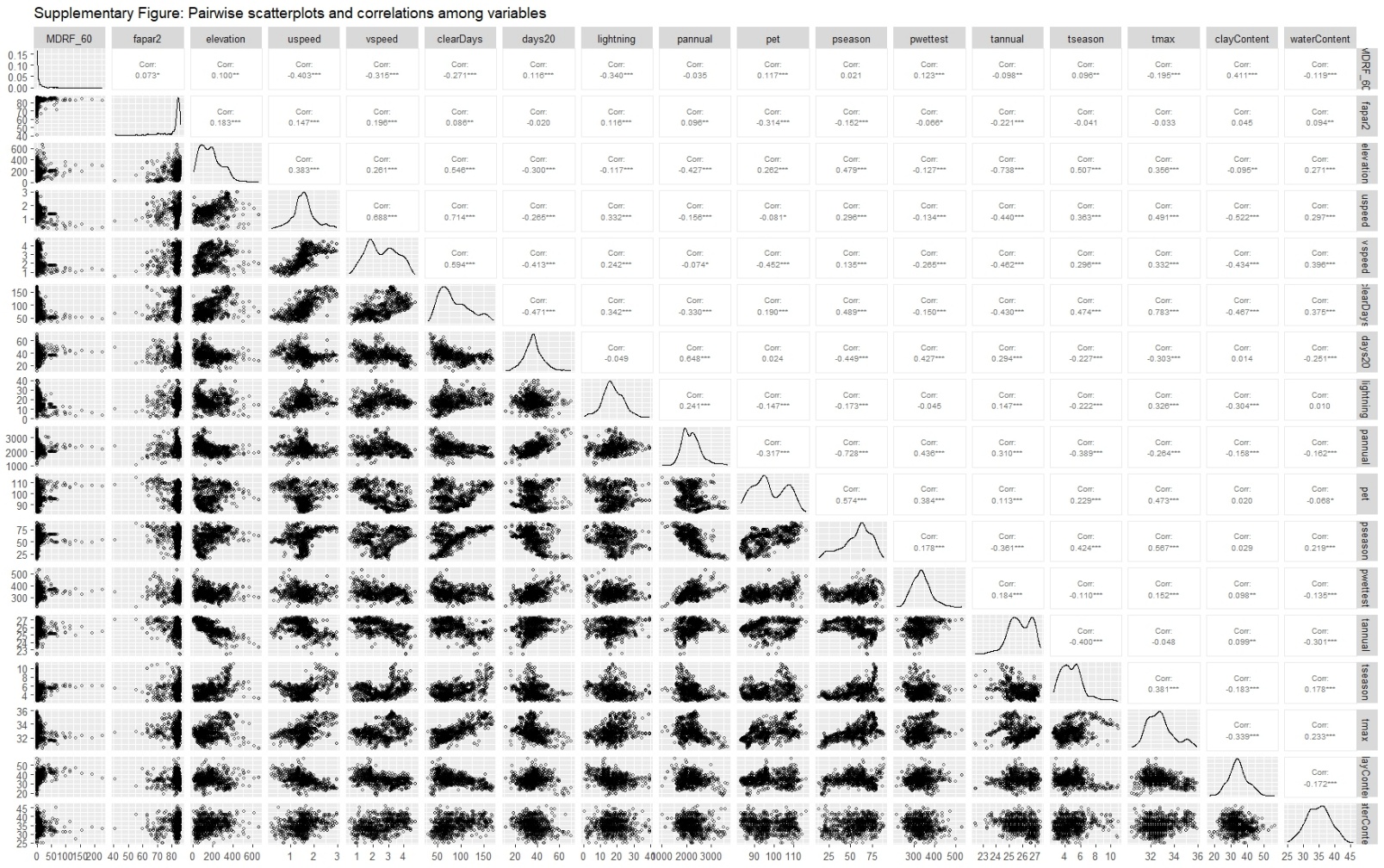


**Fig. S4** Model performance and residual diagnostics for tall tree density predictions across the Brazilian Amazon biome. (a) Relationship between observed and predicted density of tall trees (trees/km²) using the Random Forest (RF) model. Both axes are shown on a logarithmic scale. Each dot represents one of the 900 transects used in the analysis. The black line represents the linear regression fit, while the red 1:1 line represents perfect agreement. The model showed high predictive accuracy, with Pearson’s correlation coefficient R = 0.96 and root mean square error (RMSE) = 10.45. (b) Boxplot comparing the distribution of observed and predicted tree densities. The similar distribution shapes suggest that the model does not introduce systematic bias across the entire dataset, even though the RF model slightly underestimates density values per km². (c) Residual distribution (%) plotted against the predicted values, based on random cross-validation. The shaded density cloud shows the concentration of residuals, with darker areas indicating higher point density. The smooth black curve represents the trend in residuals along the range of estimated values, revealing a tendency of overestimation at low densities and underestimation at higher predicted values. Most residuals are concentrated within ±30%, especially in the low-to-intermediate prediction range. The horizontal dashed line at 0% denotes perfect prediction. (d) Spatial distribution of model residuals (%) derived from spatial cross-validation. Each point represents a transect, and colors indicate the magnitude and direction of residuals. Blue points represent areas where the model overestimated tree density, while yellow points indicate underestimation. The predominantly neutral (gray) residuals reflect a good spatial balance of predictive performance, with some localized areas showing stronger deviations, particularly in the eastern and northeastern Amazon.


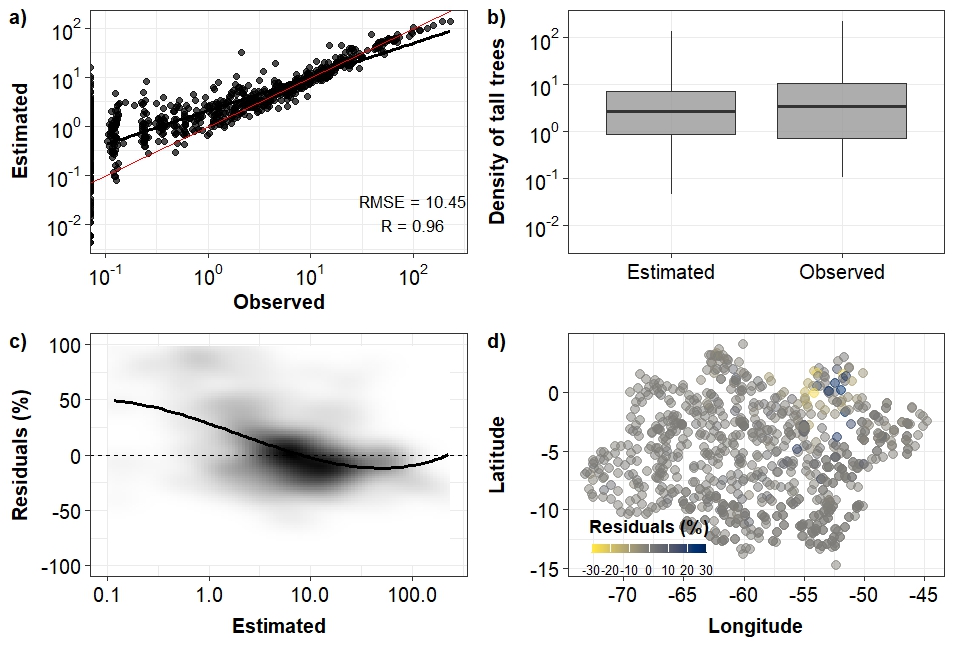


**Fig. S5** Importance of environmental predictor variables used in the Random Forest model to estimate tall tree density (⩾ 60 m) across the Amazon biome. Graph a) showcases the most critical variables, as measured by the IncNodePurity metric, underscoring the substantial influence of topographic and climatic variables. Graph b) presents the most important variables, as measured by %IncMSE, highlighting the impact of factors such as vegetation cover and water availability on the accuracy of the model generated by Random Forest. Graph a) presents the most important variables measured by the IncNodePurity metric. The IncNodePurity metric refers to the increase in node purity (i.e., the ability of a node to separate classes) of a decision tree when a variable is used to split the data. The higher the IncNodePurity value, the greater the importance of the variable in the model. The results indicate that disturbance-related variables such as uspeed and lightning, as well as soil clay content, are the most influential in predicting the density of tall trees. This suggests that variations in disturbances and clay soils play a crucial role in the distribution of these trees, directly affecting their density. Graph b) illustrates the most important variables, as measured by %IncMSE. The %IncMSE metric represents the percentage increase in the Mean Squared Error (MSE) when a variable is excluded from the model. A higher value of %IncMSE indicates that the variable is crucial to the accuracy of the model.


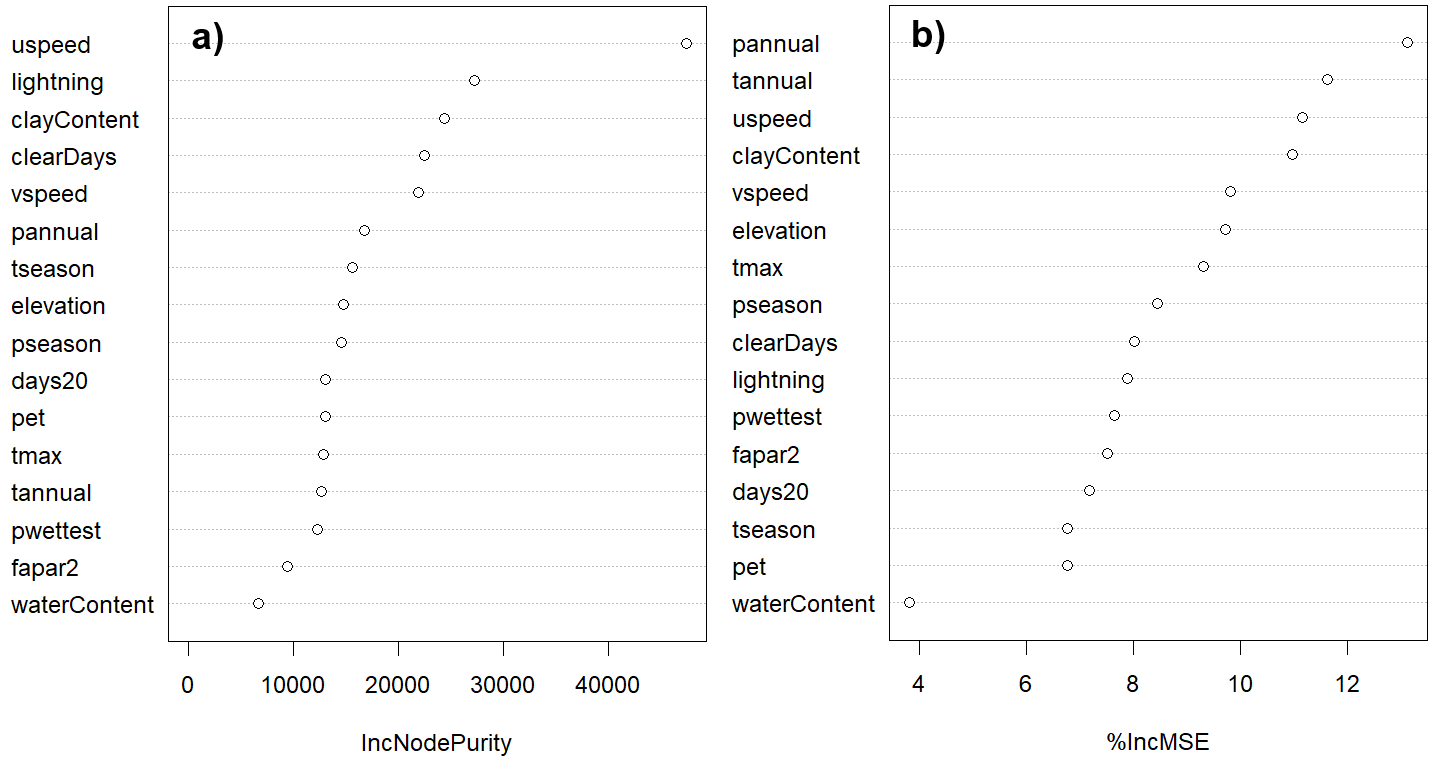


**Fig. S6** Partial dependence plot (pdp) for the environmental predictors of the Random Forest model for density of tall trees (⩾ 60 m) in the Amazon. In RF models, each predictor variable can interact with others in complex ways. The PDP is generated by calculating the average prediction of the model across all possible values of the target predictor, holding other variables fixed at their mean values. For each value of the predictor (e.g., rainfall), the model computes the average predicted response (e.g., density of tall trees), providing a smoothed curve that shows how the response varies with changes in that predictor alone.


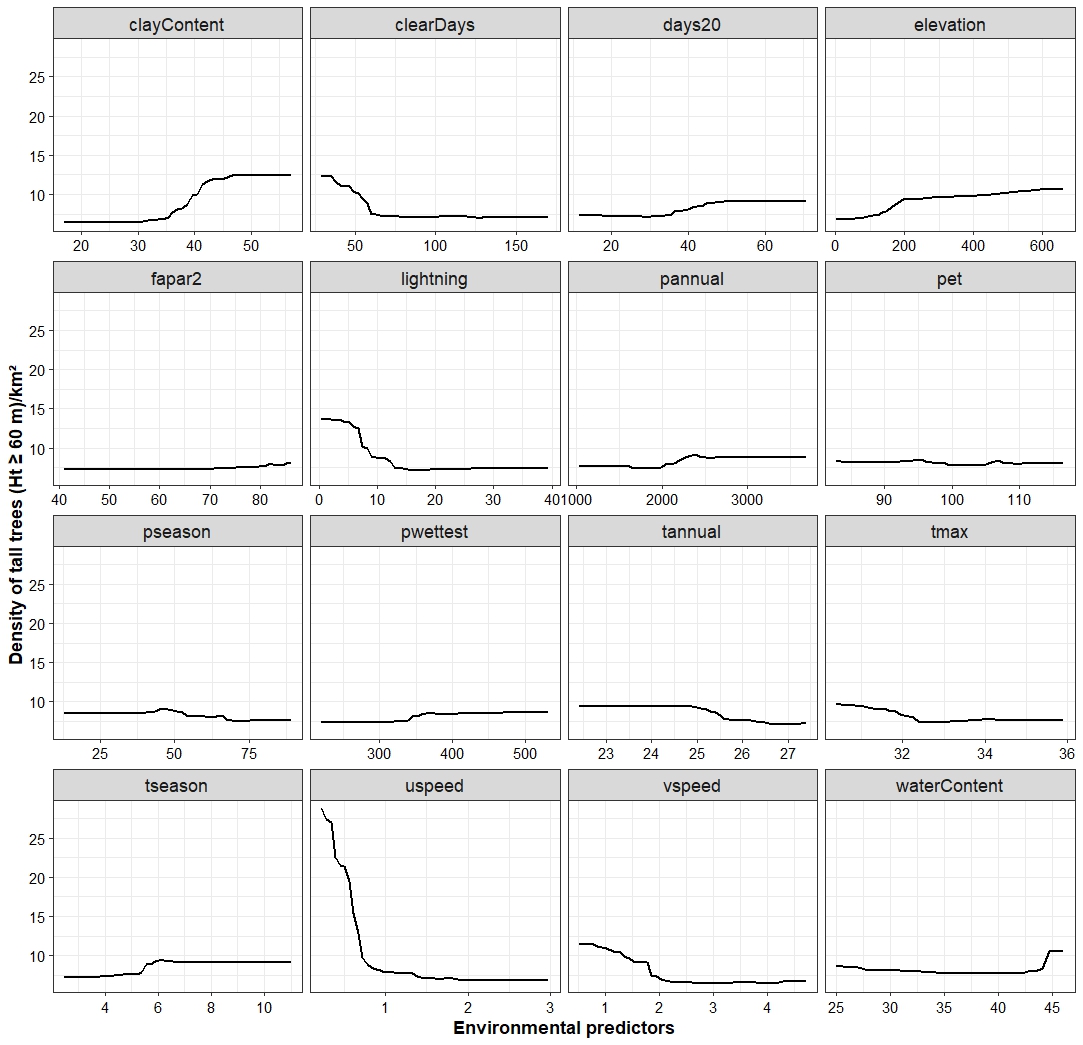


**Fig. S7** Principal components for environmental factors and biogeographic provinces: Guiana Shield (PC1 = 57.3%, PC2 = 17.6%), Pantepui (PC1 = 37.9%, PC2 = 25.9%) Roraima, (PC1 = 30.8%, PC2 = 19.8%), Imeri (PC1 = 41.4%, PC2 = 16.7%), Madeira (PC1 = 38.4, PC2 = 15.8%), Rondonia (PC1 = 42.4%, PC2 = 17.1%), Xingu-Tapajós (PC1 = 42.3%, PC2 = 15.5%), Pará (PC1 = 34.3%, PC2 = 21.6%). Gray dots represent the data samples (containing the density per km2 and the underlying environmental factors). The position of each point along PC1 and PC2 indicates the combination of variables that define that point, that is, which geographic or environmental characteristics are dominant in that sample. We observe cluster points in a specific region of the graph, which may indicate that these samples share similar environmental characteristics. These clusters may reflect common geographic areas or environmental conditions. The arrows represent the environmental variables in the graph and indicate how these variables contribute to the principal components. Variables with longer arrows are more critical for the observed variation in the data. Variables close to each other or with aligned arrows are positively correlated. Variables (arrows pointing in opposite directions) are negatively correlated. The arrows' length indicates the correlation's strength with the principal components. Longer arrows indicate variables that contribute more to the variability along the corresponding principal component. If the arrow for one variable, for example, "elevation," points to the right (positive PC1 axis) and another variable, such as "water content," points to the top (positive PC2 axis), this means that these variables are vital contributors to explaining the variability in the data.


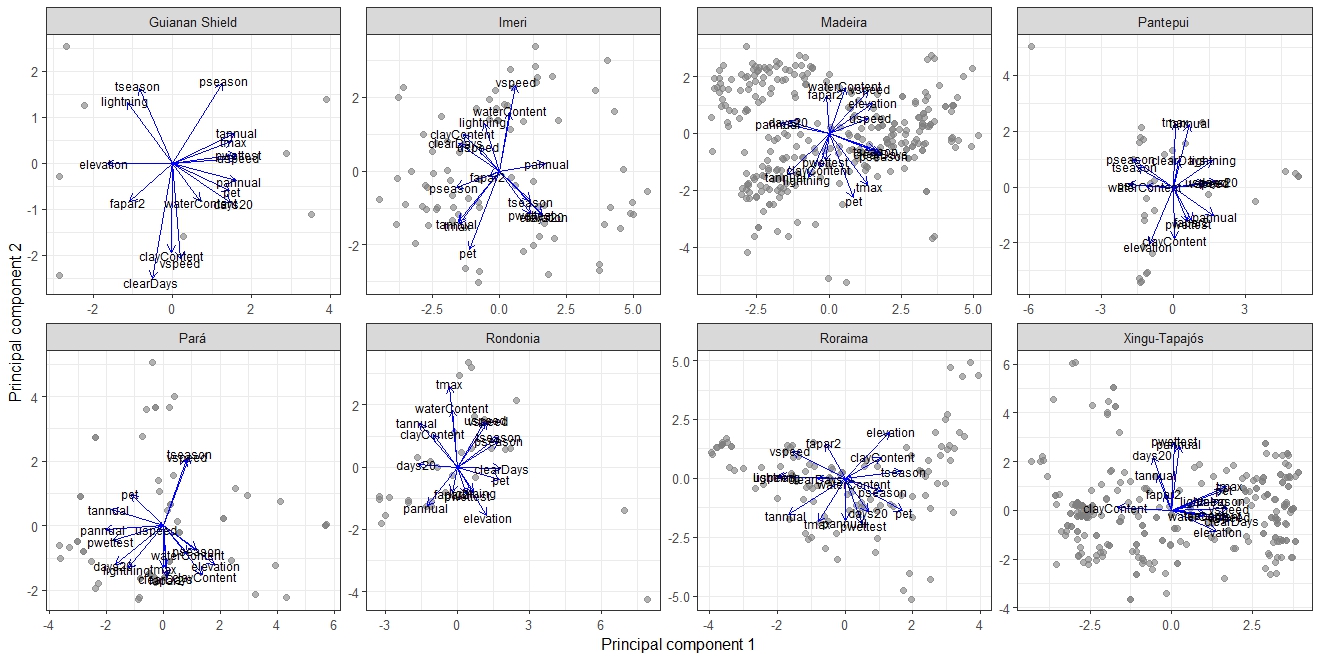


Dataset S1 (see separate Excel file). Complete dataset with the name and metrics of environmental and geographic variables, tree density, and topographic metrics generated by airborne LiDAR in 900 randomly distributed transects in the Brazilian Amazon biome.

**Notes S1** **Limitations of the study**

Firstly, linking density data with variables such as wind, lightning, solar radiation, precipitation, and soil clay and water contents can present challenges due to the complexity of ecological interactions. Models that attempt to predict density based on these variables may only capture some of the nuances of these interactions, leading to potentially inaccurate inferences (Clark, 1990).

Secondly, Fig. S4b illustrates the distribution of the tall tree density across the mapped transects. Most transects exhibit low-density values, while only a few exhibit high-density. This unbalanced sample distribution has the potential to introduce bias into model training, given the tendency of the Random Forest algorithm to be more influenced by frequent data patterns. Fig. S4b presents the relationship between observed and predicted values by the Random Forest model, demonstrating good overall predictive ability. However, it is essential to note that there are discrepancies at the extremes. The model predictions for low-density transects are more numerous, potentially leading to a lower root mean square error (RMSE) in these areas. On the other hand, the RMSE for high-density plots is likely higher due to the smaller number of samples available to represent these cases in the model. This significant sampling bias can impact the spatial model results, as areas with low density of tall trees are better represented and therefore better predicted than areas with high density. This inequality can result in a model that either underestimates or overestimates density in less-represented areas. To mitigate this effect, we consider data balancing techniques or methodological adjustments that directly address variability at different density levels through spatial cross-validation, thereby improving model robustness and accuracy. Although attempts have been made to correct density values for these sampling biases, they likely influenced, at least partially, the results.

Although the aboveground biomass (AGB) values used in this study were derived independently at the transect level from LiDAR-based observations, we acknowledge that both the AGB estimates and the tall tree density model share underlying dependencies—particularly the reliance on structural attributes derived from airborne LiDAR data. This partial methodological overlap, while not constituting circular reasoning, may contribute to the observed correlation between giant tree density and AGB by reinforcing structural relationships inherent to the forest canopy. Therefore, caution is warranted in interpreting the strength of this association as fully independent. Future efforts integrating complementary sources of biomass estimation (e.g., field plots, radar, or hyperspectral data) could help further validate and refine these relationships across biomes.

As a final point, it is crucial to highlight that different forms of land use significantly contribute to high deforestation rates, although these are not considered variables in this study. This drives reductions in the density of tall trees, especially in the southern Amazon, and can obscure the expected effects of climate, topography, and soil in several regions with tall trees. As a result, forest ecosystems are often relegated to drier regions, reversing expected within-biome relationships between moisture availability and tree density (Crowther *et al.*, 2015). Such effects may vary across biogeographic provinces, states, and municipalities depending on human population density, alternative resource availability, and socioeconomic status. Therefore, future studies should consider these factors to provide a more comprehensive understanding of the dynamics of tree density in the Amazon biome.

References

**Clark JS**. **1990**. Integration of Ecological Levels: Individual Plant Growth, Population Mortality and Ecosystem Processes. *The Journal of Ecology* **78**: 275.

**Crowther TW, Glick HB, Covey KR, Bettigole C, Maynard DS, Thomas SM, Smith JR, Hintler G, Duguid MC, Amatulli G, *et al.*** **2015**. Mapping tree density at a global scale. *Nature* **525**: 201–205.
